# Mitochondria-Targeting Polymer Micelles in Stepwise Response Releasing Gemcitabine and Destroying the Mitochondria and Nucleus for Combined Antitumor Chemotherapy

**DOI:** 10.3390/ijms232012624

**Published:** 2022-10-20

**Authors:** Shanming Zhang, Fen Zheng, Kaige Liu, Shengke Liu, Tonghu Xiao, Yabin Zhu, Long Xu

**Affiliations:** 1 Key Laboratory of Advanced Mass Spectrometry and Molecular Analysis of Zhejiang Province, School of Materials Science and Chemical Engineering, Ningbo University, Ningbo 315211, China; 2 School of Medicine, Ningbo University, Ningbo 315211, China

**Keywords:** mitochondria-targeted, pH/ROS dual-responsive, acetal, thioether, TPP

## Abstract

Mitochondrial DNA and nuclear DNA are essential genetic material which play an important role in maintaining normal metabolism, survival, and proliferation of cells. Constructing a mitochondria-targeting stimuli-responsive nano-drug delivery system releasing chemotherapeutic agents in a stepwise response manner and destroying mitochondrial DNA and nuclear DNA simultaneously is an effective way to improve the anti-tumor effect of chemotherapeutic agents. In this study, a new mitochondria-targeting pH/ROS dual-responsive block copolymer TPP-PEG2k-*b*-(BS-AA)_n_ (P1), untargeted pH/ROS dual-responsive copolymer mPEG2k-*b*-(BS-AA)_n_ (P2), pH single-responsive copolymer (mPEG2k-*b*-(AH-AA)_n_ (P3), ROS single-responsive copolymer mPEG2k-*b*-(SA-TG)_n_ (P4), and non-responsive copolymer mPEG-*b*-PCL (P5) were constructed. pH/ROS-responsive properties were characterized by proton nuclear magnetic resonance (^1^H NMR) and dynamic light scattering (DLS). Anticancer chemotherapeutic agent gemcitabine (GEM) or fluorescent substance Nile Red (NR) were loaded in the polymer micelles. Results of the mitochondrial colocalization experiment indicate that (5-carboxypentyl)(triphenyl)phosphonium bromide (TPP)-functionalized P1 micelles could be efficiently targeted and located in mitochondria. Results of the cellular uptake experiment showed that pH/ROS dual-responsive GEM-loaded P1 and P2 micelles have faster internalized and entry nucleus rates than single-responsive or non-responsive GEM-loaded micelles. The in vitro release experiment suggests pH/ROS dual-responsive GEM/P1 and GEM/P2 micelles have higher cumulative release than single-responsive GEM/P3 and GEM/P4 micelles. The in vitro cytotoxic experiment shows that the mitochondria-targeted dual-responsive GEM/P1 micelles had the lowest IC50 values, and the cytotoxic effect of dual-responsive GEM/P2 micelles was superior to the single-responsive and non-responsive drug-loaded micelles.

## 1. Introduction

Despite significant advances in medicine, materials, and nanotechnology over the past few decades, cancer still lacks effective therapeutic methods. Even worse, the incidence of different types of cancer is increasing, and cancer is the second leading cause of death worldwide [1]. Chemotherapy remains the mainstream of clinical cancer treatment [2]. Unfortunately, the severe toxic side effect and drug resistance of chemotherapeutic agents severely restrict the efficacy of cancer chemotherapy [3]. Nano-drug delivery systems can load and solubilize hydrophobic chemotherapeutic agents, which can deliver chemotherapeutic agents to tumor sites in an active (targeting receptors) or passive (EPR effect) delivery manner and improve the bioavailability and reduce the toxic side effects of chemotherapeutic agents [4,5,6]. After nanoparticles are taken up by cancer cells, their intracellular transport approach and drug release rate and release sites are key factors restricting their antitumor effects. Directly and effectively delivering chemotherapeutic agents to the intracellular site of action can significantly improve the antitumor effect of chemotherapeutic agents [7]. Tumor-specific microenvironments (high concentrations of reactive oxygen specials (ROS), glutathione, metalloproteinases, etc.) are ideal targets for stimuli-responsive nano-drug delivery systems [3]. The stimuli-responsive nano-drug delivery system precisely and rapidly releases the drug in a spatiotemporally controllable manner, significantly improving the antitumor efficacy of chemotherapeutic agents [8,9].

Precise delivery of chemotherapeutic agents to subcellular targets, such as mitochondria, nuclei, and lysosomes, is an effective way to further enhance the therapeutic effect of stimuli-responsive nanomedicine [10,11,12]. Mitochondria are the powerhouses of cells and play a very important role in cell differentiation, signal transduction, metabolism, apoptosis control, multidrug resistance development, and cancer cell metastasis [13,14]. Hence, mitochondria are ideal targets for cancer therapy [15,16]. Delivery of chemotherapeutic agents targeting mitochondria to interfere with the integrity of mitochondrial structure and function can ultimately lead to cancer cell death [17]. Furthermore, approximately 90% of intracellular ROS are generated in mitochondria, which have high concentrations of ROS [18]. Recently, ROS-responsive nano-drug delivery systems have shown great prospects and have aroused enormous enthusiasm among researchers [19]. The construction of mitochondria-targeting ROS-responsive nanodrug delivery systems is an effective way to improve the efficacy of chemotherapeutic agents.

Mitochondria have a dense and thick double-membrane structure with a high negative potential, which can effectively prevent the entry of anticancer drugs that target and act on mitochondrial [20]. The lysosome escape efficiency of nanoparticles has an important impact on the mitochondrial localization and antitumor effect of nanoparticles [21,22]. The key to improving mitochondrial localization ability and the antitumor effect of targeting mitochondria nanoparticles is to endow the nanoparticles with excellent lysosomal escape properties [22]. Delocalized lipophilic cations, especially TPP, are widely applied as mitochondria-targeted ligands to deliver chemotherapeutic drugs into mitochondria [23,24]. Many recent studies have clearly demonstrated that TPP-modified nanoparticles can efficiently escape from lysosomes [25,26,27]. Dhar et al. developed a mitochondria-targeting blended nanoparticle for the delivery of mitochondrial-acting drugs and clearly showed that conjugating TPP could effectively promote nanoparticles’ escape from lysosomes and further target and locate them in mitochondria [28]. Liu et al. reported that drug-loaded micelles modified with TPP could deliver the drug to mitochondria and reduce the capture of lysosomes [29]. However, the reported mitochondria-targeting nano-drug delivery system in order to realize multi-functionalization usually requires laborious synthesis [30] or different blended polymers [26,28], which not only bring potential security risks but are also not conducive to large-scale production. Hence, it is essential to develop mitochondria-targeting multi-functional nano-drug delivery systems with high stability, biocompatibility, and facile synthesis.

Gemcitabine is the first-line anticancer drug of many cancers; it clinically kills cancer cells by inhibiting DNA synthesis and cell growth activity [31,32]. Owing to a lack of nucleotide excision repair, mitochondrial DNA is more susceptible to damage than nuclear DNA and inhibiting the synthesis of mitochondrial DNA can lead to mitochondrial dysfunction and induce cancer cell death [33,34]. Cinnamaldehyde (CA) has an α,β-unsaturated Michael acceptor pharmacophore and exhibits potent antitumor activity by stimulating mitochondria to produce ROS and inducing oxidative stress [35]. The characteristics of low solubility and easy oxidation greatly limit the antitumor application of cinnamaldehyde. Constructing a pH or ROS-responsive nano-drug delivery system based on cinnamaldehyde and releasing cinnamaldehyde in the response to degradation is an effective method to improve the antitumor effect of cinnamaldehyde [36,37,38]. Ge et al. constructed cancer cell and mitochondrial dual-targeting nanoparticles, which exhibited good anticancer efficacy to multidrug resistance cancer cells by releasing CA toward acidic lysosomes to induce the oxidative stress of mitochondria and then accelerate the drug release rate of mitochondria-targeting nanoparticles by ROS response degradation [39].

**Scheme 1:**
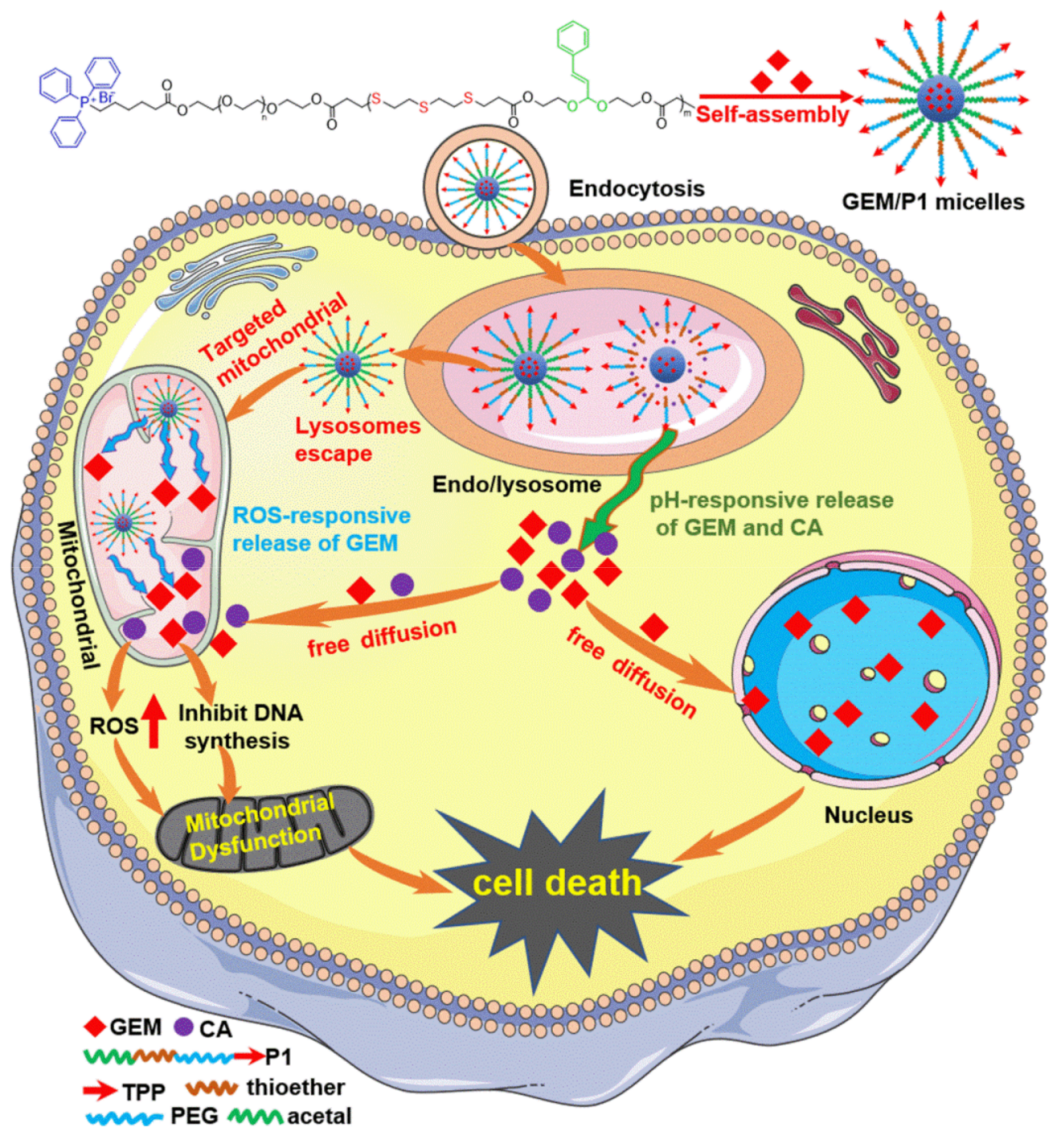
Illustration of fabrication, intracellular delivery, and responsive release behavior of GEM/P1 micelles, and the mechanism of synergistically induced cancer cell death.

In this study, we are committed to constructing a mitochondria-targeting multi-functional nano-drug delivery system with facile synthesis, high stability, and biosafety. This system will offer a delicate balance between lysosomes escape and mitochondrial localization, and then release of drugs through pH or ROS stimulation to achievethe best antitumor efficacy by destroying the mitochondria and nucleus simultaneously (Scheme 1). Once the nanoparticles are delivered to the lysosomes, some nanoparticles are degraded by acidity stimulation and release gemcitabine. Then, they synergistically induce a proton sponge effect through the protonated gemcitabine and cationic triphenylphosphine to promote the escape of remaining nanoparticles from lysosomes. The escaped nanoparticles are targeted to the mitochondria by electrostatic interaction, and then ROS-responsive release of the drug induces mitochondrial dysfunction. A double-end alkenylated acetal monomer (AA) was obtained based on cinnamaldehyde, and a mitochondria-targeted and hydrophilic end-capping reagent (TPP-PEG2k-AC) was constructed using conjugate TPP and acryloyl chloride at both ends of PEG. A novel mitochondria-targeted and pH/ROS dual-responsive block copolymer (TPP-PEG2k-*b*-(BS-AA)_n_, P1) was constructed using the Michael addition reaction. The pH/ROS responsiveness of the copolymer was studied by ^1^H NMR and DLS. The in vitro drug release properties, cellular uptake, mitochondrial colocalization, materials biosafety, and cytotoxicity of the drug-loaded micelles were studied.

## 2. Results and Discussion

### 2.1. Synthesis and Characterization of Block Copolymer

The synthetic scheme of double-end alkenylated acetal AA, capping reagent mPEG2k-AC, and TPP-PEG2k-AC is presented in Scheme S1. A new doublet peak appeared at 5.24 ppm (Figure S1), which belonged to the proton of the acetal group (-O*CH*O-). It demonstrates that acetal AA was successfully synthesized. The triplet peak that appeared at 4.32 ppm (Figure S2) was the typical characteristic peak of methylene which attached to ester bonds (-COO*CH*_2_CH_2_O-), indicating that acryloyl chloride was successfully conjugated to mPEG2k. The triplet peak that appeared at 4.17 ppm (Figure S3) was attributed to the methylene attached to ester bonds (-COO*CH*_2_CH_2_O-), and the other peaks could be well matched with the structure of TPP-PEG2k. It manifested that TPP-PEG2k was successfully obtained. All peaks that appeared in Figure S4 could be well assigned to the structure of TPP-PEG2k-AC, suggesting mitochondria-targeting hydrophilia capping reagent was prepared.

**Figure 1:**
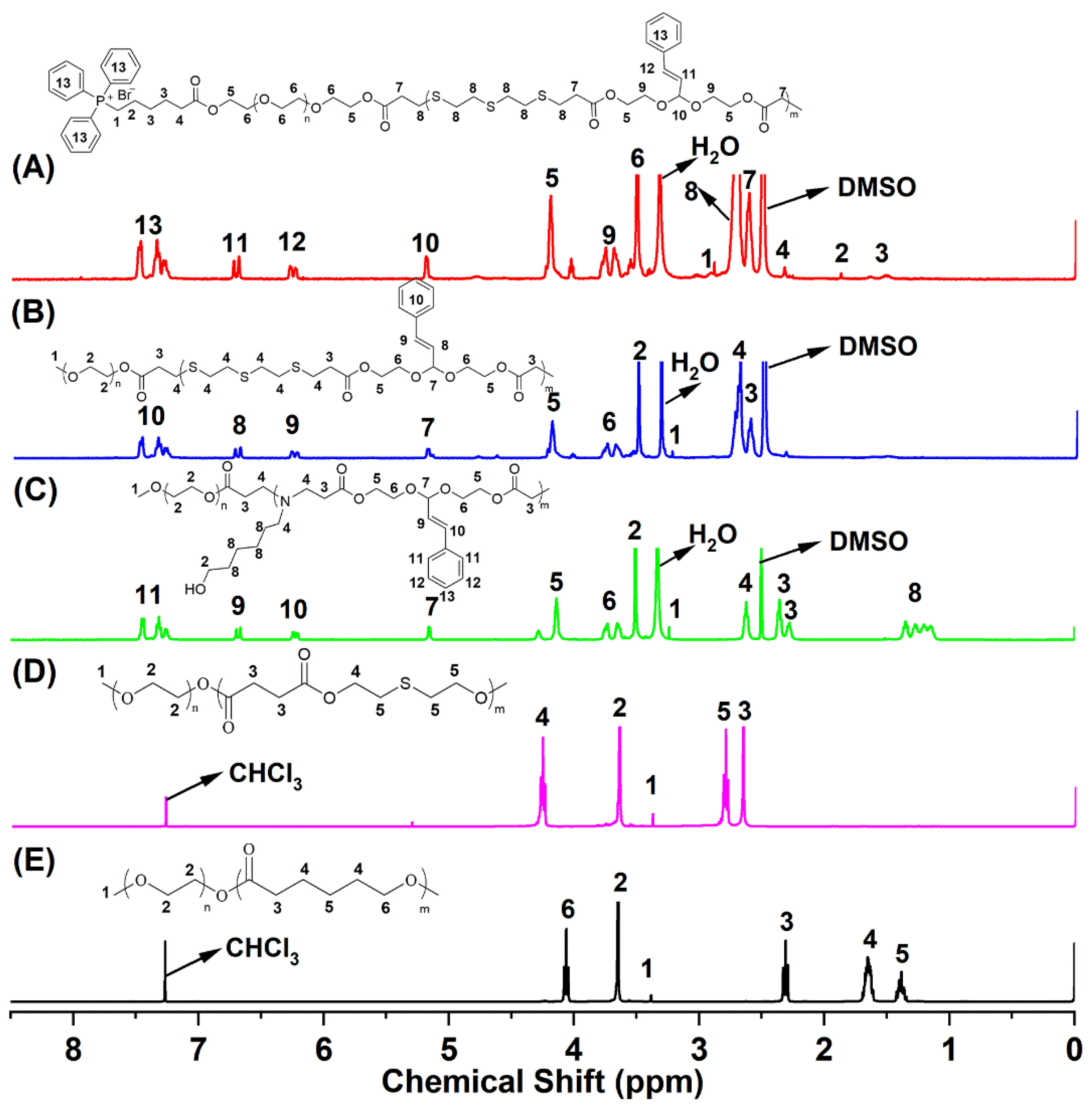
^1^H NMR spectra of mitochondria-targeting polymer TPP-PEG2k-*b*-(BS-AA)_13_ P1 (**A**), ROS/pH dual responsive polymer mPEG2k-*b*-(BS-AA)_14_ P2 (**B**), pH-responsive polymer mPEG2k-*b*-(AH-AA)_10_ P3 (**C**), ROS-responsive polymer mPEG2k-*b*-(SA-TG)_25_ P4 (**D**), and control polymer mPEG2k-*b*-PCL5k P5 (**E**).

The synthetic scheme of block copolymers P1, P2, P3, P4, and P5 is presented in Scheme S2. Peak 7 (δ 2.61 ppm, Figure 1A) is the characteristic peak of methylene which attached to carbonyl (O=C*CH*_2_CH_2_S), and the other peaks in Figure 1A could be well matched with the structure of TPP-PEG-b-(BS-AA)_n_, suggesting the Michael addition reaction proceeded successfully. Based on the ratio of peak area of peak 6 (-(*CH*_2_*CH*_2_O)_n_-) and peak 10 (-O*CH*O-), the repeating unit number of TPP-PEG-*b*-(BS-AA)_n_ (P1) was 13, and the molecule weight was 9033.06 g/mol. Peak 3 (δ 2.61 ppm, Figure 1B) was attributed to methylene proton peag which connected to carbonyl (-CO*CH*_2_CH_2_S-), and the electron-withdrawing inductive effect of the sulfur atom lead tothe chemical shift of methylene that attached to the carbonyl group shift to downfield. The results indicate that mPEG2k-*b*-(BS-AA)_n_ (P2) was successfully prepared, and the number of repeating units was 14, which was obtained by the ratio of peak area of peak 1 (*CH*_3_O-) and peak 7 (-O*CH*O-), and the molecule weight was approximately 9095.67 g/mol. Peak 4 (δ 2.62 ppm, Figure 1C) was determined as the characteristic peak of methylene which had connected to a nitrogen atom, and it suggests that the reaction proceeded successfully and the copolymer mPEG2k-*b*-(AH-AA)_n_ (P3) was obtained. According to the peak area ratio of peak 1 (*CH*_3_O-, δ 3.24 ppm) and peak 7 (-O*CH*O-, δ 5.16 ppm), the number of repeating units was 10, and the molecular weight of the P3 was approximately 6717.79 g/mol. Peak 4 (δ 4.26 ppm, Figure 1D) was attributed as the characteristic peak of a methylene proton, which was attached to the ester bond. This proves that the condensation polymerization reaction successfully proceeded, and the copolymer mPEG2k-*b*-(SA-TG)_n_ (P4) was synthesized. The number of repeating units was 25, which was obtained by comparing the ratio of the peak area of peak 1 (-O*CH*_3_) and peak 4 (-COO*CH*_2_-), and the molecule weight was approximately 7105.98 g/mol. All the peaks (Figure 1E) were attributable to mPEG-*b*-PCL (P5), which proves that the reaction was successful. The molecular weight of mPEG-*b*-PCL was consistent with the designed molecule weight (approximately 7000.00 g/mol). The ^1^H NMR spectra of copolymers P1 and P2 with a different number of repeating units were obtained by different feed ratios and are shown in Figures S5–S10. GPC was used to further characterize copolymers P1 and P2 with a different number of repeating units (Figure S11).

### 2.2. Characterization of Blank Micelles and GEM-Loaded Micelles

**Figure 2:**
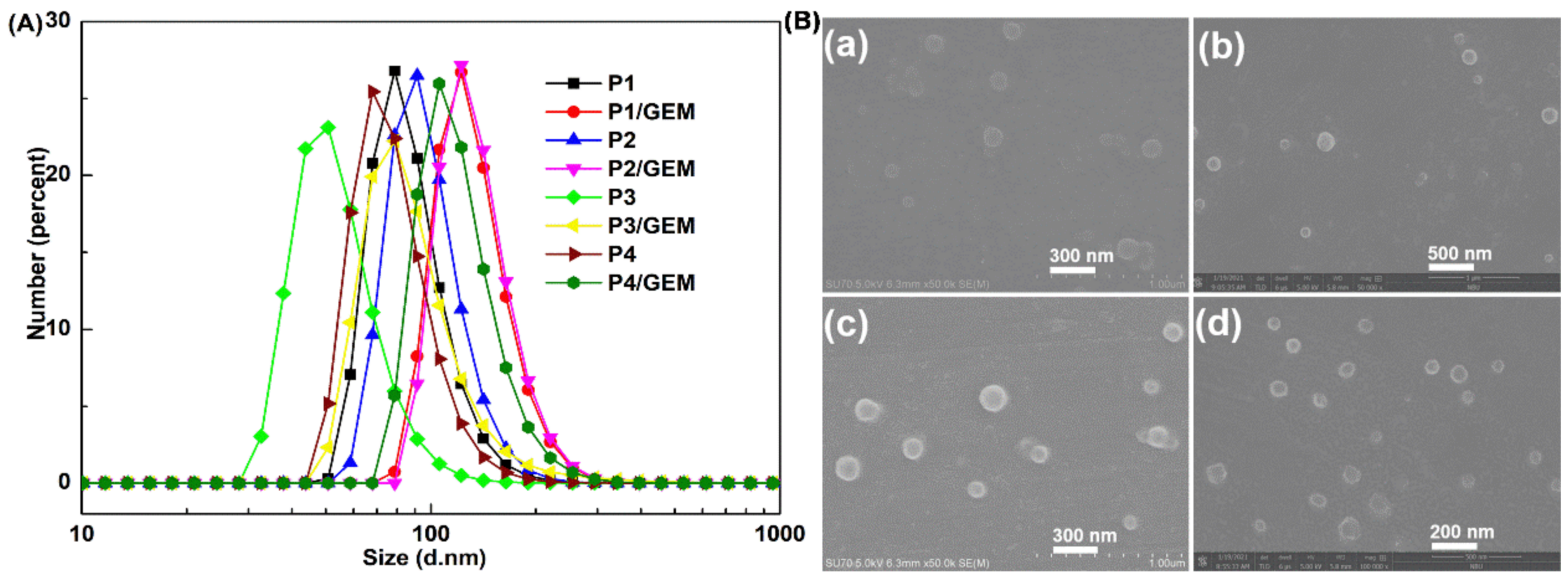
DLS results (**A**) of blank and GEM-loaded P1, P2, P3, and P4 micelles; SEM images (**B**) of blank P1 micelles (**a**), GEM/P1 micelles (**b**), blank P2 micelles (**c**), and GEM/P2 micelles (**d**).

The size and size distribution of blank and GEM-loaded polymeric micelles was studied by DLS, and the results are presented in Figure 2A. The size and polydispersity index (PDI) of blank P1, P2, P3, and P4 micelles were 87.71 nm (PDI = 0.112), 97.9 nm (PDI = 0.150), 54.8 nm (PDI = 0.194), and 78.91 nm (PDI = 0.168), respectively. The size and PDI of GEM-loaded P1, P2, P3, and P4 micelles were 133.5 nm (PDI = 0.041), 136.1 nm (PDI = 0.219), 92.6 nm (PDI = 0.101), and 121.2 nm (PDI = 0.142), respectively. The size of GEM-loaded micelles increased significantly compared to the corresponding blank micelles. SEM was further employed to observe the size and morphology of blank and GEM-loaded P1 and P2 micelles. Both blank and GEM-loaded micelles were spherical nanoparticles (Figure 2B). Compared to the results of DLS, the size observed by SEM was similar or smaller. Since the nanoparticles observed by SEM were dry and dehydrated, the drying process may lead to the shrinking of micelles [40].

The CMC of copolymers P1, P2, P3, and P4 was measured using a pyrene method. CMC reflects the stability of the micelles. The smaller the CMC value, the stronger the anti-dilution ability of the micelles and the more stable the micelles. A copolymer with a low CMC value is favorable to maintain the integrity of the drug-loaded micelle in the in vivo drug delivery process. The CMCs of P1, P2, P3, and P4 were 9.03, 7.03, 8.96, and 6.93 µg/mL (Figure S12), respectively. The CMCs of P1 and P2 with a different number of repeating units are presented in Figure S13 and Figure S14, respectively. Stability is a key index for evaluating the performance of micelles. After one week of incubation in neutral PBS solution at room temperature, the size and PDI of the polymer P1 and P2 micelles with a different number of repeating units showed slight changes (Figure S15). The results prove that polymer P1 and P2 micelles with different numbers of repeating units have high stability.

Nanoprecipitation method was selected to prepare blank micelles and GEM-loaded micelles. The DLCs of GEM/P1, GEMP2, GEM/P3, GEM/P4, and GEM/P5 measured by ultraviolet spectroscopy were 12.32%, 10.15%, 9.83%, 8.92%, and 4.52%, respectively. The EEs of these GEM-loaded micelles were 61.6%, 51.1%, 48.65%, 47.15%, and 22.5%, respectively.

### 2.3. pH/ROS-Responsiveness

**Figure 3:**
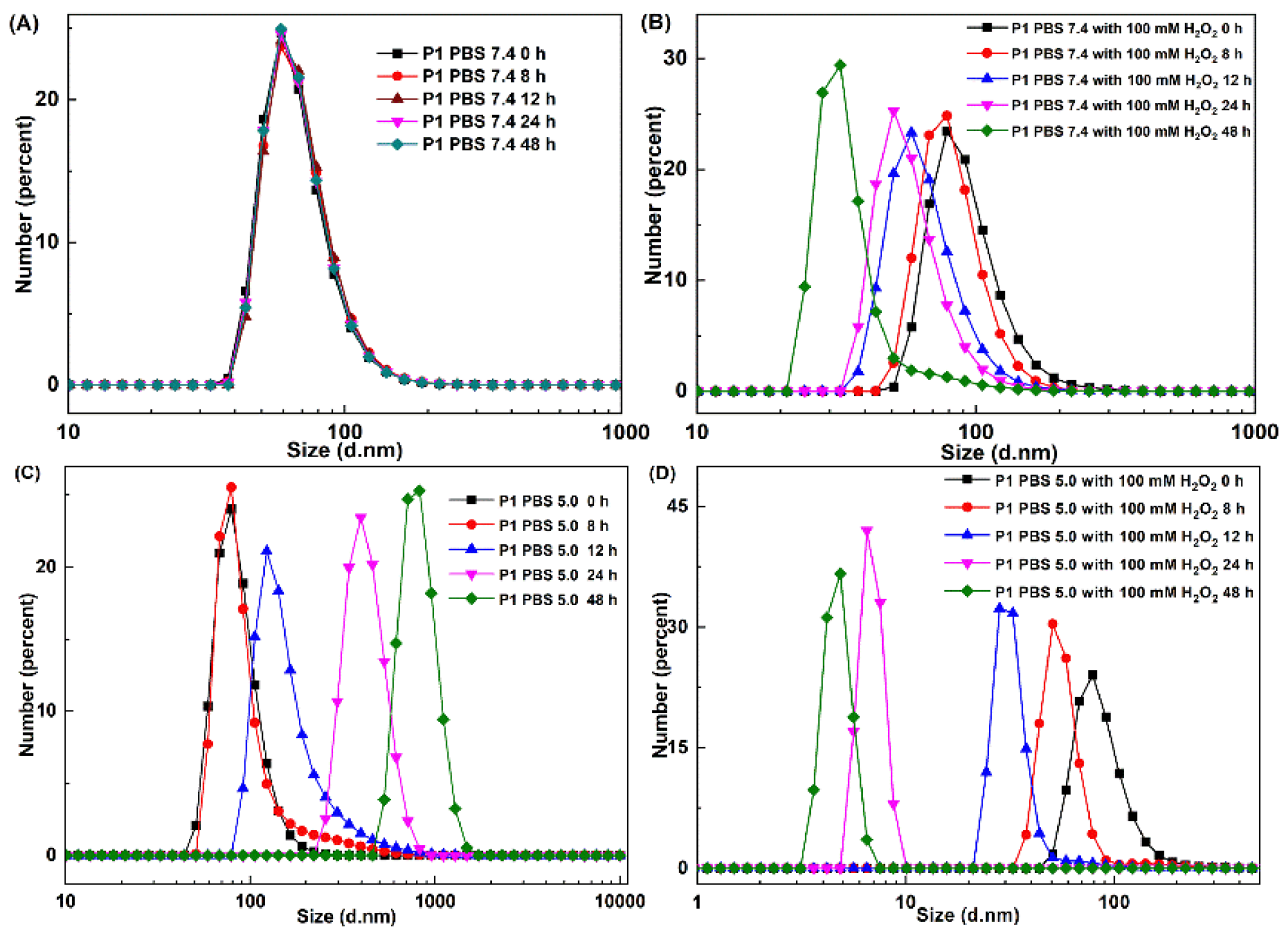
Size changes of TPP-PEG2k-*b*-(BS-AA)_13_ P1 micelle incubated with PBS 7.4 (**A**), PBS 7.4 with 100 mM H_2_O_2_ (**B**), PBS 5.0 (**C**), and PBS 5.0 with 100 mM H_2_O_2_ (**D**) for different times.

Changes in micelle sizes under ROS and pH conditions were used to investigate the ROS-responsive and pH-responsive properties of micelles, respectively. Blank P1 micelles exhibited high stability in neutral PBS solution (Figure 3A) and showed responsiveness both in ROS (Figure 3B) and acidic environments (Figure 3C). The size of P1 micelles decreased from the initial 90 nm to approximately 10 nm after being treated with 100 mM H_2_O_2_ for 48 h (Figure 3B). The results indicate that the hydrophobic thioether structure of the P1 micelle was oxidized to hydrophilic sulfoxide or sulfone and led to the dissociation of micelles. The size of P1 micelles increased significantly after being treated in acidic conditions for 12 h, and the size increased to 800 nm after being incubated in an acid environment for 48 h (Figure 3C). The increase in particle size indicates that the micelles swelled under acidic conditions, which may be caused by gradually undergoing responsive cleavage of the acetal structure under acidic conditions. Under an acid H_2_O_2_ environment, micelles exhibited faster response speed, and a significant particle size reduction could be observed after incubation for 8 h. Furthermore, the particle size decreased to approximately 5 nm after being treated for 48 h (Figure 3D). This suggests that an acidic H_2_O_2_ environment can accelerate the rupture of P1 micelles through pH-responsive-induced swelling and ROS-responsive-induced dissociation. P2 micelles without a mitochondria-targeting group exhibited pH and ROS-responsive properties similarly to P1 micelles (Figure S16). In addition, those containing acetal structure P3 micelles were observed to increase in size after incubation under acidic conditions (Figure S17), and those containing thioether structure P4 micelles were observed to significantly decrease in particle size under H_2_O_2_ conditions (Figure S18). This indicates that the P3 micelles have pH responsiveness, and the P4 micelles have ROS responsiveness.

**Figure 4:**
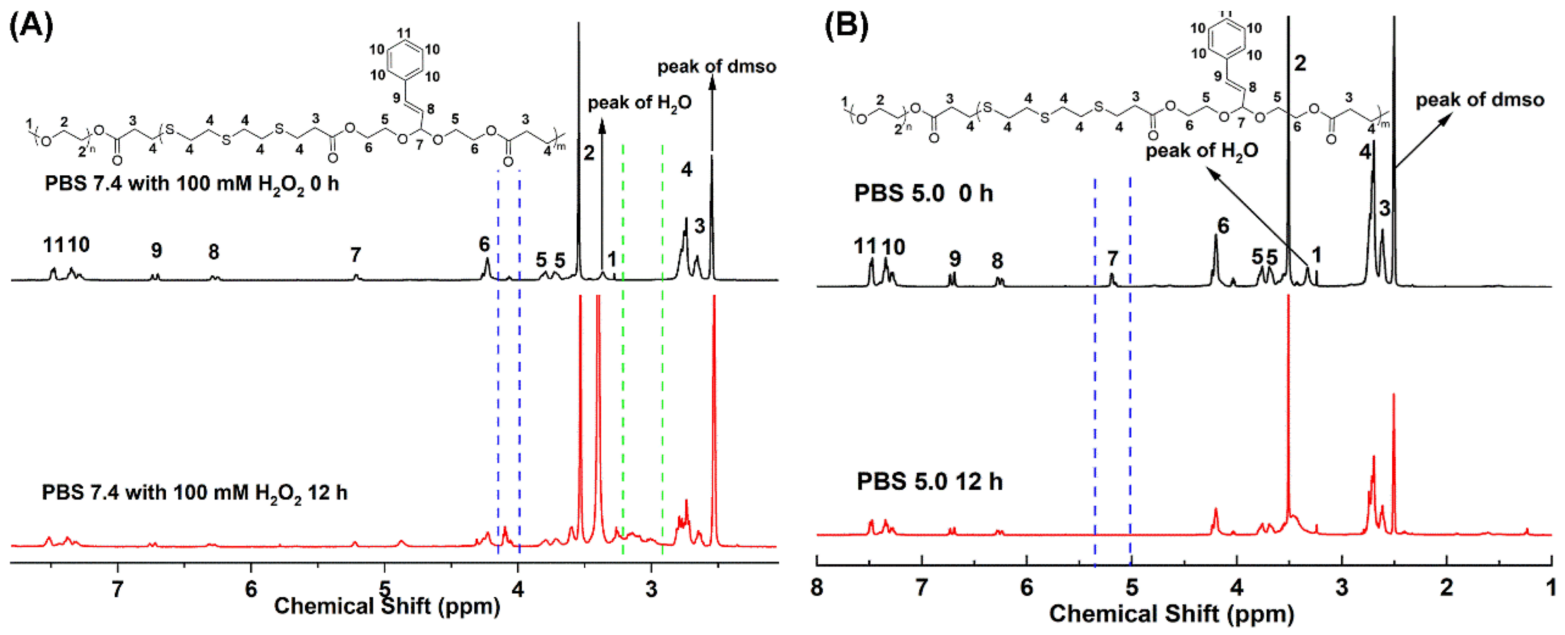
^1^H NMR spectra of mPEG2k-*b*-(BS-AA)_14_ P2 micelle before and after incubated with containing 100 mM H_2_O_2_ neutral PBS solution (**A**) and acid PBS solution (**B**) for 12 h.

Containing acetal and thioether structures, P2 micelles were selected to further study the structural changes after being treated with acidic or ROS conditions by ^1^H NMR spectroscopy. New peaks appeared at 3.00, 3.13, and 4.10 ppm (Figure 4A), suggesting the structure of thioether was oxidized to the structure of sulfoxide and sulfone, which has a stronger electron-withdrawing inductive effect than sulfur atoms and leads to the chemical shift of methylene which is adjacent to the sulfur atom shift to downfield [41]. The results declare that the polymer micelle containing thioether moieties has ROS responsiveness. The typical acetal proton peak (peak 7) disappears after being treated under an acid environment (Figure 4B), which demonstrates that the acetal moieties in polymer micelles have been hydrolyzed. It manifests that the polymer micelle containing acetal moieties has pH responsiveness.

### 2.4. In Vitro Drug Release

**Figure 5:**
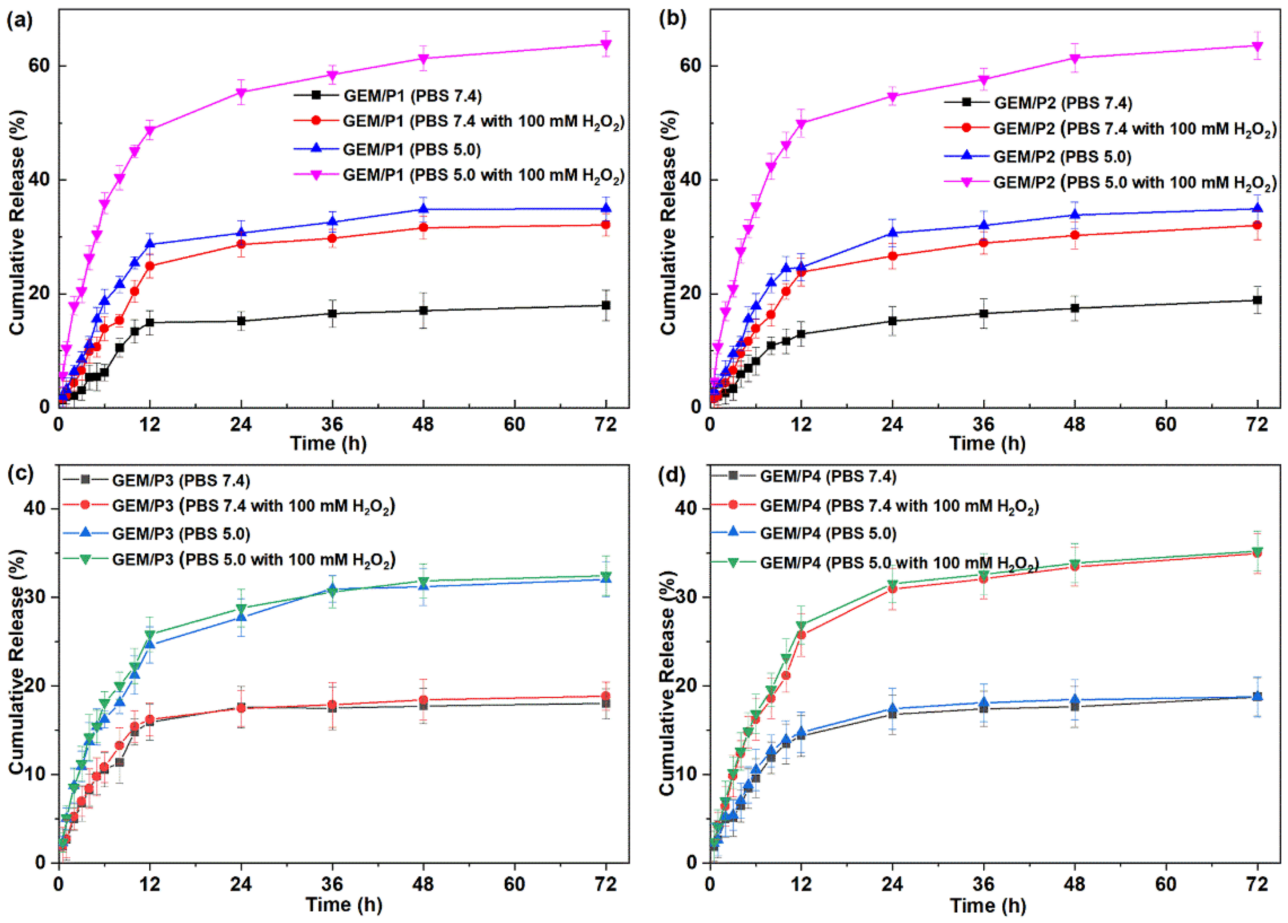
Drug release profiles of GEM/P1 micelle (**a**), GEM/P2 micelle (**b**), GEM/P3 micelle (**c**), and GEM/P4 micelles (**d**) treated with neutral PBS (pH 7.4), acid PBS (pH 5.0), neutral PBS (7.4) with 100 mM H_2_O_2_, and acid PBS (5.0) with 100 mM H_2_O_2_.

In order to evaluate the response performance and drug release properties of drug-loaded micelles in the physiological environment and tumor microenvironment, neutral PBS (pH 7.4), acid PBS (pH 5.0), PBS (7.4) with 100 mM H_2_O_2_, and PBS (5.0) with 100 mM H_2_O_2_ at 37 °C were chosen for in vitro release experiments. After incubation in neutral and acidic environments for 72 h, the cumulative release rates of GEM/P1 micelles were 17.99% and 32.12%, respectively (Figure 5A). The results show that GEM/P1 micelles were quite stable in neutral conditions and presented accelerated release under acidic conditions, indicating that GEM/P1 micelles have pH-responsive release properties. Compared with the result of incubation in a neutral environment, the cumulative release of GEM/P1 was increased after being treated with a neutral H_2_O_2_ solution, and the cumulative release was 34.96% (Figure 5A), suggesting that the GEM/P1 micelles have ROS-responsive release properties. GEM/P1 micelles had the fastest release performance in an acidic H_2_O_2_ solution, and the cumulative release was 63.91% after incubation for 72 h (Figure 5A). The data indicate that GEM/P1 micelles could accelerate the release of drugs through pH- and ROS-responsive degradation. The cumulative release rates of GEM/P2 micelles were 18.90%, 32.04%, 34.94%, and 63.61% after being treated with neutral PBS (pH 7.4), acid PBS (pH 5.0), PBS (7.4) with 100 mM H_2_O_2_, and PBS (5.0) with 100 mM H_2_O_2_ for 72 h (Figure 5B). GEM/P2 micelles without the mitochondria-targeting group exhibited comparable in vitro release behaviors with those containing mitochondria-targeting group GEM/P1 micelles. The cumulative release rates of GEM/P3 micelles under neutral solution, neutral H_2_O_2_ solution, acidic solution, and an acidic H_2_O_2_ environment for 72 h were 18.01%, 18.86%, 32.12%, and 32.48% (Figure 5C), respectively. The result shows that containing acetal moieties GEM/P3 micelles had pH-sensitive drug release properties. Containing a thioether structure, GEM/P4 micelles exhibited ROS-responsive and pH-inert drug release properties, and the cumulative release rates was 18.76%, 18.78%, 34.97%, and 35.24% after being treated with neutral solution, neutral H_2_O_2_ solution, acidic solution, and an acidic H_2_O_2_ environment for 72 h (Figure 5D).

### 2.5. Colocalization in Mitochondria

**Figure 6:**
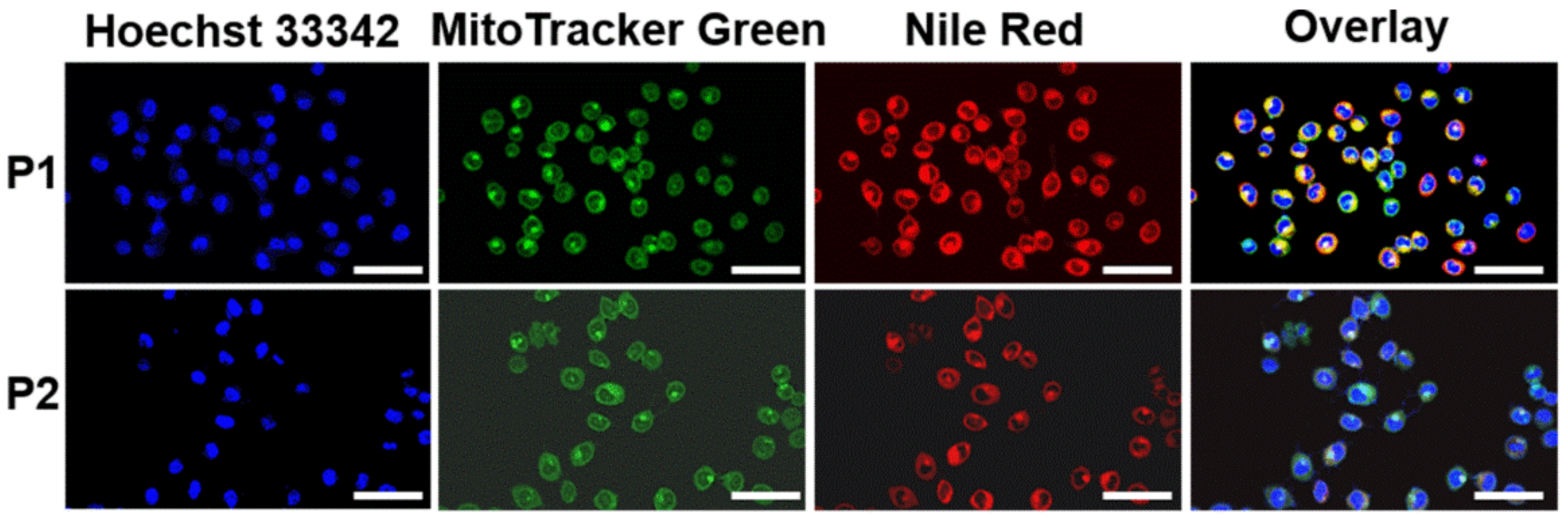
Colocalization of P1 micelles with TPP group and P2 micelles without TPP group in mitochondria. Skov3 cells were treated with NR-loaded micelles for 4 h and stained with Hoechst 33,342 and Mito-Tracker Green FM for observation. The scale bar was 50 µm.

A confocal laser scanning fluorescence microscope was applied to observe the mitochondria-targeting properties of the drug-loaded P1 micelles in Skov3 cells. NR, a model drug with strong red fluorescence, instead of gemcitabine, was used to prepare NR-loaded micelles to study the mitochondrial localization properties of drug-loaded P1 micelles [42]. The nuclei and mitochondria of Skov3 cells were sequentially labeled with blue fluorescent Hoechst 33,342 and green fluorescent Mito-Tracker Green FM [43]. Yellow fluorescence was observed in the mitochondria in the overlay images of cells treated with NR/P1 micelles (Figure 6), meaning that NR/P1 micelles entered the mitochondria. The green mitochondria fluorescence and the red Nile Red fluorescence were superimposed to form yellow fluorescence. However, yellow fluorescence did not appear in the mitochondria in the overlay images of cells incubated with P2 micelles for 4 h (Figure 6). P2 micelles without TPP groups cannot target and locate in mitochondrial. These results suggest conjugating TPP on the surface of nanoparticles could lead to effective lysosome escape and mitochondrial localization. After the nanoparticles were internalized and transported to the lysosome, a small fraction of the nanoparticles showed pH-responsive degradation and released hydrophobic gemcitabine, which was subsequently protonated. Protonated gemcitabine with cationic TPP synergistically induces a proton sponge effect and facilitates the successful escape of the remaining nanoparticles from the lysosome, targeting the mitochondria by electrostatic interaction [28,29,30].

### 2.6. Cellular Uptake

**Figure 7:**
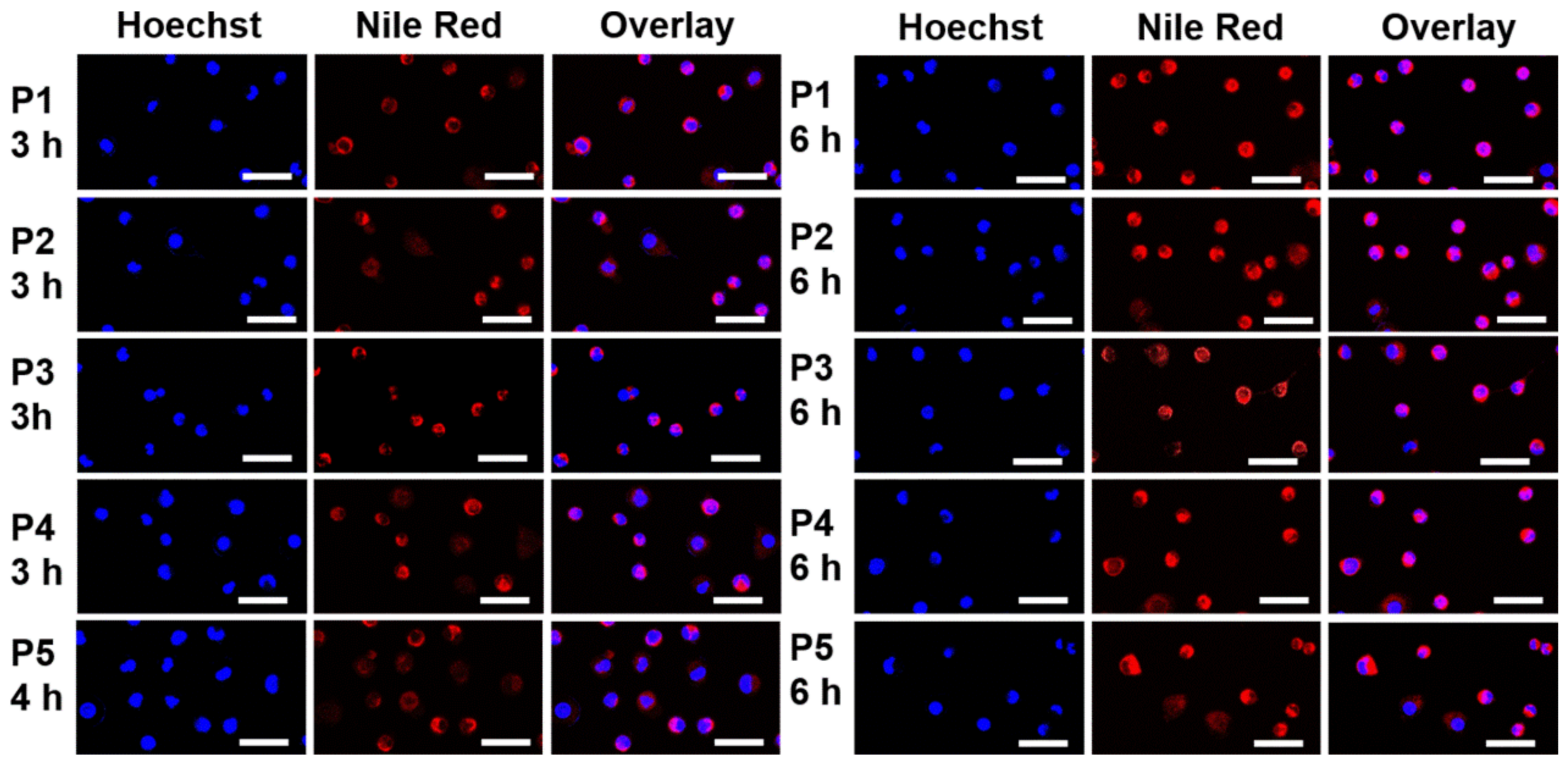
CLSM images of Skov3 cells treated with NR-loaded P1, P2, P3, P4, and P5 micelles at different times, and the cell nucleus was stained with Hoechst 33342. The scale bar is 50 µm.

To study the internalized and intracellular distribution of drug-loaded micelles, hydrophobic NR was encapsulated into P1, P2, P3, P4, and P5 micelles. Red fluorescence was observed in the cytoplasm after incubation for 3 h, which illustrated that drug-loaded micelles could be rapidly taken up by cells (Figure 7). As the incubation time increased to 6 h, more intense red fluorescence was observed in cancer cells, and red fluorescence appeared in the nucleus, especially in the cells treated with NR-loaded P1 and P2 micelles (Figure 7). In order to show the difference in fluorescence intensity more clearly, ImageJ software (1.48, National Institutes of Health, America) was used to conduct semi-quantitative analysis of CLSM images. Dates of fluorescence intensity were analyzed using Prism 4 (Graph Pad Prism 5 Software, San Diego, CA, USA) and expressed as mean ± standard (SD) deviation. Statistical significance (*p* < 0.05) was evaluated using One-way ANOVA. The cells treated with pH/ROS dual-responsive mitochondria-targeting NR/P1 micelles exhibited the strongest mean fluorescence intensity (Figure S19). Cells treated with pH/ROS dual-responsive NR/P2 micelles showed superior mean fluorescence intensity than the cells treated with single-responsive and non-responsive micelles (Figure S19). The mitochondria-targeting NR/P1 micelles have the fastest relative rate of entry into the cell and nucleus. The electrostatic interaction of TPP conjugating P1 micelles with cytomembrane may accelerate micelles’ internalization. Intracellular micelles quickly release NR by pH/ROS dual-responsive degradation, leading to NR’s rapidly entering the nucleus. The pH/ROS dual-responsive NR/P2 micelles exhibited superior cell uptake properties compared to single-responsive and non-responsive NR-loaded micelles. This was possible because the pH/ROS dual-responsive micelles could release NR faster via dual-responsive degradation, leading to a high concentration of drug outside the nucleus, then entering into the nucleus faster by freely diffusing.

### 2.7. Cytotoxicity

**Figure 8:**
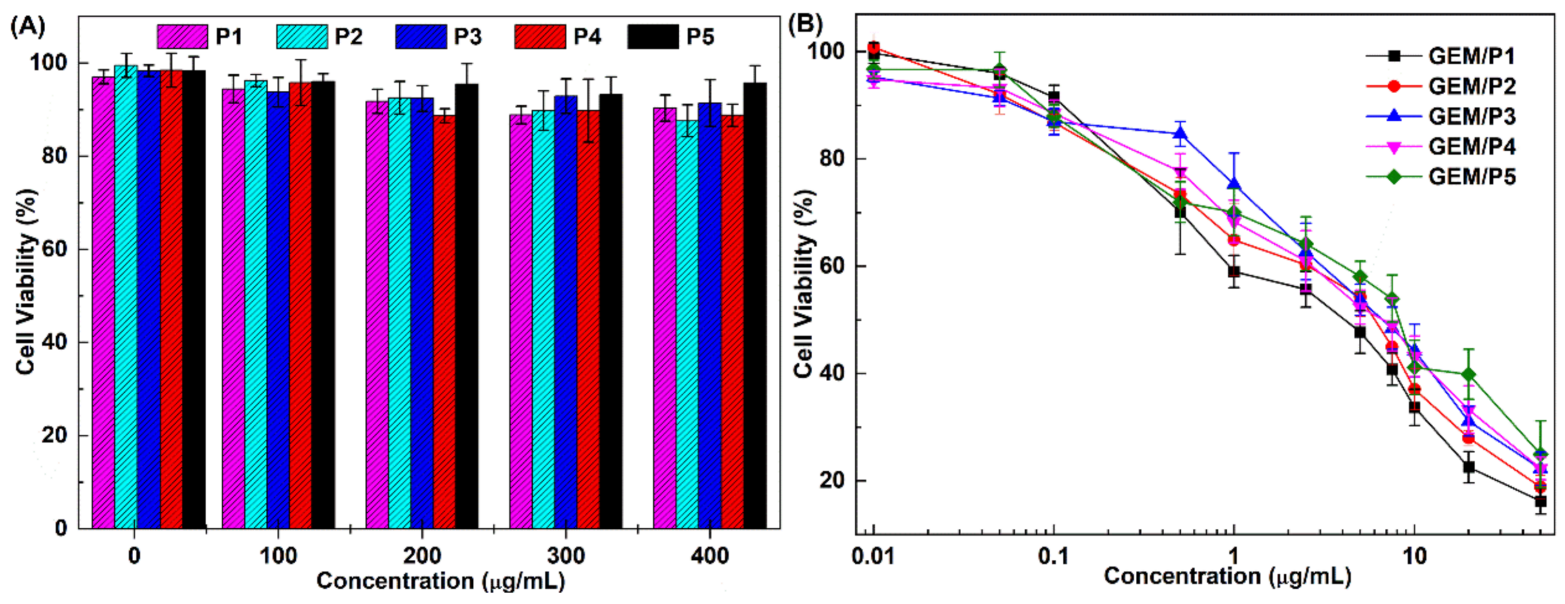
Cell viability of Skov3 cells (**A**) after incubated with different concentrations of P1, P2, P3, and P4 micelles for 48 h, and cell viability of Skov3 cells (**B**) after incubated with GEM-loaded micelles for 72 h. The results are shown as mean ± SD, *n* = 3.

Biocompatibility of polymer P1, P2, P3, P4, and P5 micelles was evaluated by an MTT assay in Skov3 cells. The survival rate of Skov3 cells was higher than 90% after incubation for 48 h, even at a high micelle concentration of 400 µg/mL (Figure 8A). These results indicate that all polymer micelles were biocompatible.

In vitro cytotoxic experiments of GEM-loaded micelles against Skov3 cells were studied. Half maximal inhibitory concentration (IC50) values of GEM/P1 micelles, GEM/P2 micelles, GEM/P3 micelles, GEM/P4 micelles, and GEM/P5 micelles against Skov3 cells were 4.15, 6.05, 6.74, 6.76, and 8.28 μg/mL, respectively (Figure 8B). GEM/P1 micelles displayed the lowest IC50 values, which may be caused by the following reasons. Firstly, TPP conjugating P1 micelles can facilitate the pH-responsive release of cinnamaldehyde and gemcitabine and promote lysosome escape and mitochondrial targeting. Secondly, cinnamaldehyde and gemcitabine can separately freely diffuse into mitochondria and the nucleus to induce mitochondrial dysfunction and cell apoptosis. Thirdly, CA stimulates mitochondria to generate ROS and contributes to maintaining a high concentration of ROS within mitochondria, which accelerates the ROS-responsive release of gemcitabine and inhibits mitochondrial DNA synthesis from inducing mitochondrial dysfunction and cancer cell apoptosis. Lastly, GEM/P1 micelles have fastest response release rates and good performance of nuclear entry among all drug-loaded micelles (it can be supported by the results of in vitro drug release and cellular uptake experiment), which can effectively inhibit the synthesis of nuclear DNA and induce cancer cell apoptosis. The IC50 value of GEM/P2 micelles without the mitochondrial targeting group was lower than GEM/P3, GEM/P4, and GEM/P5 micelles. This was probably because GEM/P2 micelles could release drugs faster in a pH/ROS dual-responsive manner compared to single-responsive GEM/P3 and GEM/P4 micelles and non-responsive GEM/P5 micelles. The pH single-responsive GEM/P3 micelles and ROS single-responsive GEM/P4 showed comparable antitumor activity. GEM/P5 micelles had the biggest IC50 values, which was probably due to GEM/P5 micelles being pH/ROS-inert, meaning they could not accelerate the release of the drug by response degradation.

## 3. Methods and Materials

### 3.1. Materials

All solvents and reagents used in this study were chemically pure. Cinnamaldehyde (CA), N,N′-carbonyldiimidazole (CDI), methylthiazoletetrazolium (MTT), and 1,8-diazabicycloundec-7-ene (DBU) were purchased from Saen chemical technology Co., Ltd. (Shanghai, China) Methoxy poly(ethylene glycol) (mPEG2k, *M*_w_ = 2000 g/mol) and Poly(ethylene glycol) (PEG2k, *M*_w_ = 2000 g/mol) were purchased from Sigma Aldrich Co., Ltd. (Shanghai, China) Acryloyl chloride (AC), 6-amino-1-hexanol (AH), 2-hydroxyethyl acrylate, and bis(2-mercaptoethyl) sulfide (BS) were purchased from Aladdin Bio-Chem technology Co., Ltd. (Shanghai, China) Thiodiglycol (TG) was provided by Chengdu Huaxia Chemical Reagent Co., Ltd. (Chengdu, Sichuan Province) Nile Redwas bought from Yuanye Biotechnology Co., Ltd. (Shanghai, China) Gemcitabine hydrochloride, hydrogen peroxide, (5-carboxypentyl)(triphenyl)phosphonium bromide, succinic acid (SA), and other solvents were purchased from Shanghai Titan Technology Co., Ltd. (Shanghai, China) Mito-Tracker Green FM was purchased from Beyotime Biotechnology Co., Ltd. (Shanghai, China) Dulbecco’s modified Eagle’s medium (DMEM), Roswell Park Memorial Institute (RPMI) 1640 medium, and fetal bovine serum (FBS) were purchased from Energy Chemical Co. (Shanghai, China).

### 3.2. Characterizations

The chemical structure of small molecules and polymers was characterized by proton nuclear magnetic resonance (^1^H NMR) spectroscopy using tetramethylsilane as the internal standard (Bruker Avance II NMR spectrometer, 500 MHz). The molecular weight of the copolymers was determined by gel permeation chromatography (GPC, Agilent GPC PL50) using DMF as the eluent at a flow rate of 1.0 mL/min and polystyrene (PS) as the standard. The average size and size distribution of the polymer micelles were measured by a dynamic light scattering (DLS) spectrometer (Malvern Zetasizer Nano ZS, Malvern, UK). A scanning electron microscope (SEM, Magellan400, Hillsboro, OR, USA) was employed to observe the morphology of the nanoparticles. Ultraviolet-visible spectroscopy (TU-1950, Persee, Shanghai, China) was applied to measure the drug loading content. Fluorescence spectroscopy (F-7100, Hitachi High-Technologies, Hitachi, Japan) was studied to determine the critical micelle concentration and drug-releasing amount.

### 3.3. Synthesis of Double-End Alkenylated Acetal (AA)

A catalytic amount of p-toluenesulfonic acid (0.01 g, 0.006 mmol) and anhydrous toluene (30 mL) were added to cinnamaldehyde (1.50 mL, 12 mmol) and stirred at room temperature for 2 h. Then, hydroxyethyl acrylate (3.78 mL, 36 mmol) and 1,4-hydroquinone (0.01 g, 1 mmol) were added, and reflux reaction was carried out for 24 h at 135 °C. The reaction-generated water was then removed through the water separator to promote the forward progress of the reaction. Subsequently, a few drops of triethylamine (TEA) were added to quench the reaction. The reaction solution was concentrated by rotary evaporation. The product was obtained by column chromatography using petroleum ether and ethyl acetate (10:1) as the eluent.

### 3.4. Synthesis of Acryloyl Chloride Conjugated Methoxy Poly(ethylene glycol) (mPEG2k-AC)

mPEG2k (3 g, 1.5 mmol) was added to a side-neck flask and vacuumed at 105 °C for 3 h to remove water. After cooling to room temperature (RT), dried dichloromethane (25 mL) was added to dissolve it. Subsequently, the temperature was lowered to 0 °C, acryloyl chloride (0.61 mL, 7.5 mmol) and dried triethylamine (1.04 mL, 7.5 mmol) were slowly added, and the mixture was reacted for 72 h at RT under nitrogen protection. The reaction solution was washed three times with saturated sodium bicarbonate solution, dried with anhydrous sodium sulfate, and concentrated in the reaction solution. The concentrated solution was precipitated in cold ether three times. The remaining solid was collected and filtrated, then dried to obtain mPEG2k-AC.

### 3.5. Synthesis of Triphenylphosphine Conjugated Poly(ethylene glycol) (TPP-PEG2k)

PEG2k (3.0 g, 1.5 mmol) was added to a side-necked flask and dried by vacuum for 3 h at 105 °C. TPP (1.37 g, 3 mmol) was dissolved in 25 mL dry chloroform, and CDI (0.93 g, 6 mmol) was added to activate the carboxyl, then the activated solution was carefully injected into the dried PEG and continuously stirred for 72 h at 45 °C under the protection of nitrogen. After completing the reaction, the reaction solution was concentrated, precipitated three times in cold ether, collected the solid by filtration, and dried to obtain the product (TPP-PEG2k).

### 3.6. Synthesis of Acryloyl Chloride Conjugated TPP-PEG2k (TPP-PEG2k-AC)

The synthetic method of alkenylated TPP-PEG2k was similar to the synthesis of alkenylated methoxy poly(ethylene glycol).

### 3.7. Synthesis of pH/ROS Dual-Responsive Block Copolymer Targeting Mitochondria (TPP-PEG2k-b-(BS-AA)_n_)

Mitochondria-targeting block copolymers were prepared by the Michael addition reaction. Three block copolymers with different hydrophilic and hydrophobic ratios were prepared according to different feeding ratios (1:20:20, 1:30:30, and 1:40:40). The equivalent relationship of TPP-PEG2k-AC, AA, and BS was taken as 1:30:30 as an example to illustrate the synthesis method. TPP-PEG2k-AC (0.25 g, 0.125 mmol) was added to a side-neck flask and dried by vacuum for 3h at 105 °C. BS (0.49 mL, 3.75 mmol), AA (1.3 g, 3.75 mmol), and DBU (1 mL, 7.5 mmol) were dissolved in 25 mL of dried DMSO, then the mixed solution was quickly injected into the dry and cooled TPP-PEG2k-AC, and was continuously stirred for 72 h at 45 °C under nitrogen atmosphere. Subsequently, the reaction solution was concentrated and dropwise added to 200 mL of deionized water under stirring. After stirring for 12 h, it was transferred to a dialysis bag with a molecular weight cut-off of 2000 for dialysis. The water was changed every 6 h, and dialysis was conducted for 72 h. The solution of dialysis was centrifuged at 3000 rpm/min for 5 min, and the supernatant was collected and freeze-dried to obtain the product (mPEG2k-*b*-(BS-AA)_n_).

### 3.8. Synthesis of pH/ROS Dual-Responsive Block Copolymers (mPEG2k-b-(BS-AA)_n_)

The synthetic method of control material without a mitochondrial targeting group was similar to the syntheses of TPP-PEG2k-*b*-(BS-AA)_n_.

### 3.9. Synthesis of pH-Responsive Block Copolymers (mPEG2k-b-(AH-AA)_n_)

mPEG2k-AC (0.25 g, 0.125 mmol) was added to a side-neck flask and dried by vacuum for 3 h at 105 °C. AA (0.85 g, 2.5 mmol), AH (0.30 g, 2.5 mmol), and DBU (0.75 mL, 5 mmol) were dissolved in 25 mL of dried DMSO, the mixed solution was quickly moved into the dried mPEG2k-AC, and the solution was stirred for 72 h at 45 °C under the protection of N_2_. Then, the reaction solution was concentrated and dropwise added to 200 mL of deionized water under stirring. After stirring for 12 h, the solution was transferred to a dialysis bag with a molecular weight cut-off of 2000 and for dialysis for 72 h, during which the water was changed every 6 h. The solution in the dialysis bag was centrifuged at 4000 rpm/min for 5 min, and the supernatant was collected and lyophilized to obtain an amphiphilic copolymer (mPEG2k-*b*-(AH-AA)_n_).

### 3.10. Synthesis of ROS-Responsive Block Copolymers (mPEG2k-b-(SA-TG)_n_)

mPEG2k (1.25 g, 0.25 mmol) and TG (1.53 g, 12.5 mmol) were added to a side-neck flask and dried by vacuum for 3 h at 105 °C. SA (1.48 g, 12.5 mmol) and CDI (4.46 g, 27.5 mmol) were added to a round-bottomed flask, followed by 30 mL of dried DMSO and stirring at room temperature for 0.5 h. The activated solution was injected into dried mPEG2k and thiodiglycol and reacted for 2 h at 60 °C under nitrogen protection. The reaction solution was concentrated and dropwise added to 200 mL of deionized water under stirring for 12 h. Then, the solution was transferred into a dialysis bag with a molecular weight cut-off of 2000 and dialysis for 72 h, during which the water was changed every 6 h. The solution in the dialysis bag was centrifuged at 3000 rpm/min for 5 min, and the supernatant was collected and lyophilized to obtain an amphiphilic copolymer (mPEG2k-*b*-(SA-TG)_n_).

### 3.11. Synthesis of Non-Responsive Block Copolymers

mPEG2k (2.5 g, 0.5 mmol) and caprolactone (5.0 g, 43.8 mmol) were placed in a long-neck polymerization tube and dried by vacuum for 3 h at 95 °C. After cooling to room temperature, a catalytic amount of stannous octoate toluene solution was added. The toluene was removed by vacuum at RT. Then, the tube was sealed and immersed in an oil bath and was reacted for 72 h at 130 °C. The mixture was dissolved in dichloromethane and precipitated in cold ethyl ether three times. mPEG2k-*b*-PCL5k was obtained after drying under vacuum overnight.

### 3.12. Preparation of Blank Micelles and GEM-Loaded Micelles

The blank polymeric micelles and GEM-loaded micelles were prepared by the nanoprecipitation method. In detail, 10 mg of amphiphilic block polymer were dissolved in 1 mL of DMSO by ultrasonication. Then, the solution was dropwise added into 10 mL of deionized water under strong agitation. After stirring for 12 h, the solution was transferred into a dialysis bag and the DMSO was removed by dialysis in deionized water. The blank micelle solution was obtained after lyophilization. In total, 2.5 mg GEM were dissolved in 2 mL DMSO, and then 25 μL dry triethylamine were added and stirred at room temperature for 12 h. Subsequently, 10 mg of the polymer were added and stirred for 4 h. The solution was then dropwise added to 7 mL deionized water under stirring and continuously stirred inthe dark for 12 h. Then, it was transferred into a dialysis bag and the DMSO was removed by dialysis in deionized water. The solution was then centrifuged at 3000 rpm/min for 5 min, and the supernatant was freeze-dried to obtain the GEM-loaded micelles.

### 3.13. Critical Micelle Concentration

A pyrene fluorescence probe method was used to determine the critical micelle concentration (CMC) of polymers. In short, the freshly prepared micelle solution was diluted to different concentrations with a volume of 1 mL, and then 10 μL of pyrene acetone solution (6 × 10^−5^ M) were added to the different concentrations of micelle solution. The solutions were mixed evenly by vortex and the acetone was volatilized at RT, then the excitation spectra (emission wavelength: 390 nm) of the micelle solution with different concentrations was measured. The ratio of the fluorescence intensity at 384 nm to the fluorescence intensity at 373 nm under different micelle concentrations was recorded and calculated. In the diagram of the relationship between the ratio (I384/I373) and concentration, the concentration corresponding to the inflection point was CMC.

### 3.14. Drug-Loading Content

Drug loading contents (DLCs) of GEM in GEM-loaded micelles were measured by ultraviolet-visible absorption spectrum using the standard curve method (detection wavelength is 285 nm). A series of variant concentrations of free GEM in DMSO and H_2_O (*v*/*v*, 9:1) and the absorbance value were measured to obtain the standard curve. The absorbance value of the GEM-loaded micelle was determined similarly. DLC and encapsulation efficiency (EE) were calculated by the following formulas:

DLC (%) = (weight of drug determined by ultraviolet spectrum/weight of drug-loaded micelle) × 100%,

EE (%) = (weight of drug determined by ultraviolet spectrum/weight of feeding drug) × 100%.

### 3.15. ROS/pH-Responsiveness

For ^1^H NMR study: ^1^H NMR spectra were applied to characterize the ROS/pH-responsiveness of the polymer by investigating the structural changes of the polymer after the treatment of H_2_O_2_ or acid PBS solution. In total, 100 mM H_2_O_2_ solution were obtained by adding 0.1 mL H_2_O_2_ (30%) to 9.9 mL of PBS (pH 7.4). Polymeric micelle solution (5 mg/mL, 4 mL) was mixed with H_2_O_2_ (100 mM, 4 mL) or acid PBS solution (pH 5.0, 4 mL) by vortex. The solution was then incubated at 37 °C for 24 h. The solution was lyophilized and characterized by ^1^H NMR.

For DLS study: The changes in micellar size in ROS/pH condition and PBS (pH 7.4) were investigated by DLS to verify the ROS/pH-responsiveness of polymeric micelles. Specifically, 0.5 mL blank micelle (1 mg/mL) were mixed with 0.5 mL of ROS reagent or 0.5 mL acid PBS solution (pH 5.0) and incubated at 37 °C in a constant temperature water bath oscillator. A blank micelle in PBS (pH 7.4) was used as a control. The micellar size was recorded by DLS at the predetermined time interval.

### 3.16. In Vitro Drug Release

Neutral PBS (pH 7.4) with or without H_2_O_2_ and acid PBS (5.0) with or without ROS reagent (H_2_O_2_) were chosen to simulate the physiological environment and tumor environment to study the in vitro drug release properties of GEM-loaded polymeric micelles. Then, 1 mL of freshly prepared GEM-loaded micelles was transferred to a dialysis bag (molecular weight cut-off of 2 kD), the ends were tied with cotton string, and the dialysis bag was transferred to a screw-thread bottle containing 25 mL of different release media. The screw bottle was placed in a constant temperature water bath oscillator, and the in vitro release experiment was carried out at 37 °C. Subsequently, 1 mL of release solution was taken off at predetermined time points, and 1 mL fresh medium was added. The GEM concentration of the release solution was measured by fluorescence spectrometry and the cumulative release was calculated. Three parallel experiments were performed for each sample.

### 3.17. Cellular Uptake

Confocal laser scanning microscopy (CLSM) was used to investigate the cellular uptake and intracellular distribution of drug-loaded micelles using skov3 cells. NR was chosen as the model drug for cellular tracer uptake and intracellular distribution of drug-loaded micelles. In total, 1 mL of cell suspension solution (1 × 10^3^ cells per mL) was added to 35 mm diameter glass dishes and incubated for 24 h. Then, the culture medium was replaced with a fresh culture medium containing NR-loaded micelles, and the final NR concentration was 10 μg/mL. After incubation for 3 or 6 h, the culture medium was removed, and 300 μL of Hoechst 33,342 staining solution (20 μg/mL) were added. The cells were incubated for 15 min and washed with PBS three times, covered with 300 μL of PBS, and instantly observed by CLSM.

### 3.18. Co-Localization in Mitochondria

CLSM was applied to observe the enrichment and localization of mitochondria-targeting micelles in mitochondria. Skov3 cells were grown on glass dishes and incubated for 24 h, then treated with NR-loaded micelles for 4 h. Subsequently, the NR-containing solutions were removed, and 300 μL of Hoechst 33,342 staining solution (20 μg/mL) were added. After incubating for 15 min, the staining solution was removed and washed with PBS three times. The cells were then incubated with MitoTracker Green FM for 30 min, washed with PBS, and observed instantly by CLSM.

### 3.19. Biocompatibility Experiment

The biocompatibility of the synthesized polymers was tested by an MTT assay using Skov3 cells. In detail, cells were seeded in 96-well plates with a density of 5 × 10^3^ cells per well and incubated for 24 h. The culture medium was replaced with a fresh, completed culture medium containing different concentrations of the polymeric micelle. After incubating the medium for an additional 48 h, the culture medium was removed; then, 90 µL blank culture medium and 10 µL MTT solution (5 mg/mL) were added into each well. After incubation for 3 h, the culture medium containing MTT was removed, and 100 µL DMSO were added to dissolve the obtained formazan crystals. The absorbance was measured by a microplate reader at the wavelength of 490 nm, and the results were shown as average ± SD (*n* = 3).

### 3.20. In Vitro Cytotoxicity Experiment

The in vitro cytotoxicity experiment of the GEM-loaded micelles against Skov3 cells was studied by an MTT assay. In short, 100 μL of cell suspension solution were added into 96-well plates with a density of 1 × 10^5^ cells per well and incubated for 24 h. Then, the culture medium was replaced with different concentrations of GEM-loaded micelle completed culture medium. After co-incubation for 72 h, cell viability was determined via an MTT assay, as mentioned previously.

## 4. Conclusions

Mitochondria-targeting pH/ROS dual-responsive copolymer TPP-PEG2k-*b*-(BS-AA)_n_ (P1), pH/ROS dual-responsive copolymer mPEG2k-*b*-(BS-AA)_n_ (P2), pH single-responsive copolymer mPEG2k-*b*-(AH-AA)_n_ (P3), ROS single-responsive copolymer mPEG2k-*b*-(SA-TG)_n_ (P4), and non-responsive copolymer mPEG—*b*-PCL (P5) were synthesized. The pH/ROS-responsiveness of polymeric micelles (P1–P4) was demonstrated by ^1^H NMR or DLS. Containing acetal and thioether moieties, P1 and P2 micelles exhibited the fastest response speeds and the largest cumulative release under acidic ROS conditions. TPP functional P1 micelles were efficiently localized in mitochondria. Responsive micelles rapidly entered cells and released the drug to diffuse into the nucleus, especially containing acetal and thioether structure P1 and P2 micelles. Mitochondria-targeting pH/ROS dual-responsive GEM/P1 micelles had the lowest IC50 values. This reveals that mitochondria-targeting and pH/ROS stepwise response copolymer TPP-PEG2k*-b*-(BS-AA)_n_ is a promising carrier for anticancer drug delivery.

## Supplementary Materials

The supporting information can be downloaded at: www.mdpi.com/article/10.3390/ijms232012624/s1.
