# Supplementary material for: Mitochondria-Targeting Polymer Micelles in Stepwise Response Releasing Gemcitabine and Destroying the Mitochondria and Nucleus for Combined Antitumor Chemotherapy
Source: Int J Mol Sci. 2022 Oct 20;23(20):12624. doi: 10.3390/ijms232012624 (PMC9604266; doi:10.3390/ijms232012624)
Supplement: Supplementary file 1 [file ijms-23-12624-s001.zip › ijms-1921144-supplementary.pdf]

Mitochondria-targeting polymer micelles stepwise response releasing gemcitabine destroying mitochondria and nucleus for combined antitumor chemotherapy

Shanming Zhang[a], Fen Zheng[a], Kaige Liu[a], Shengke Liu[a], Tonghu Xiao[a], Yabin Zhu[b] and Long Xu[a]*

[a]Key Laboratory of Advanced Mass Spectrometry and Molecular Analysis of Zhejiang Province, School of Materials Science and Chemical Engineering, Ningbo University, Ningbo, 315211, China

[b]School of medicine, Ningbo University, Ningbo 315211, China.

* Corresponding author, E-mail: xulong@nbu.edu.cn

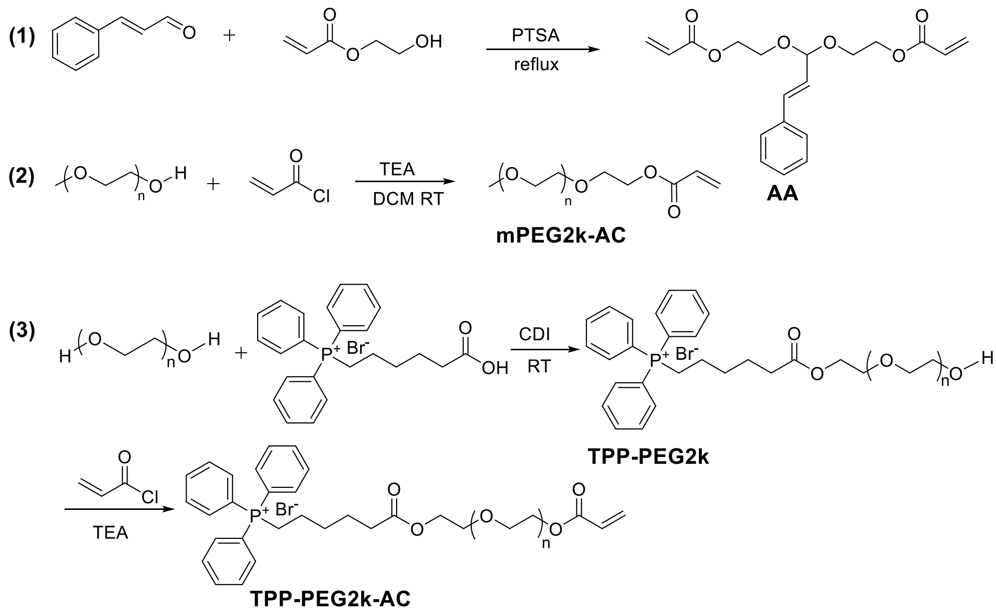

Scheme S1. Synthetic scheme of double-end alkenylated acetal AA, capping reagent mPEG2k-AC and TPP-PEG2k-AC.

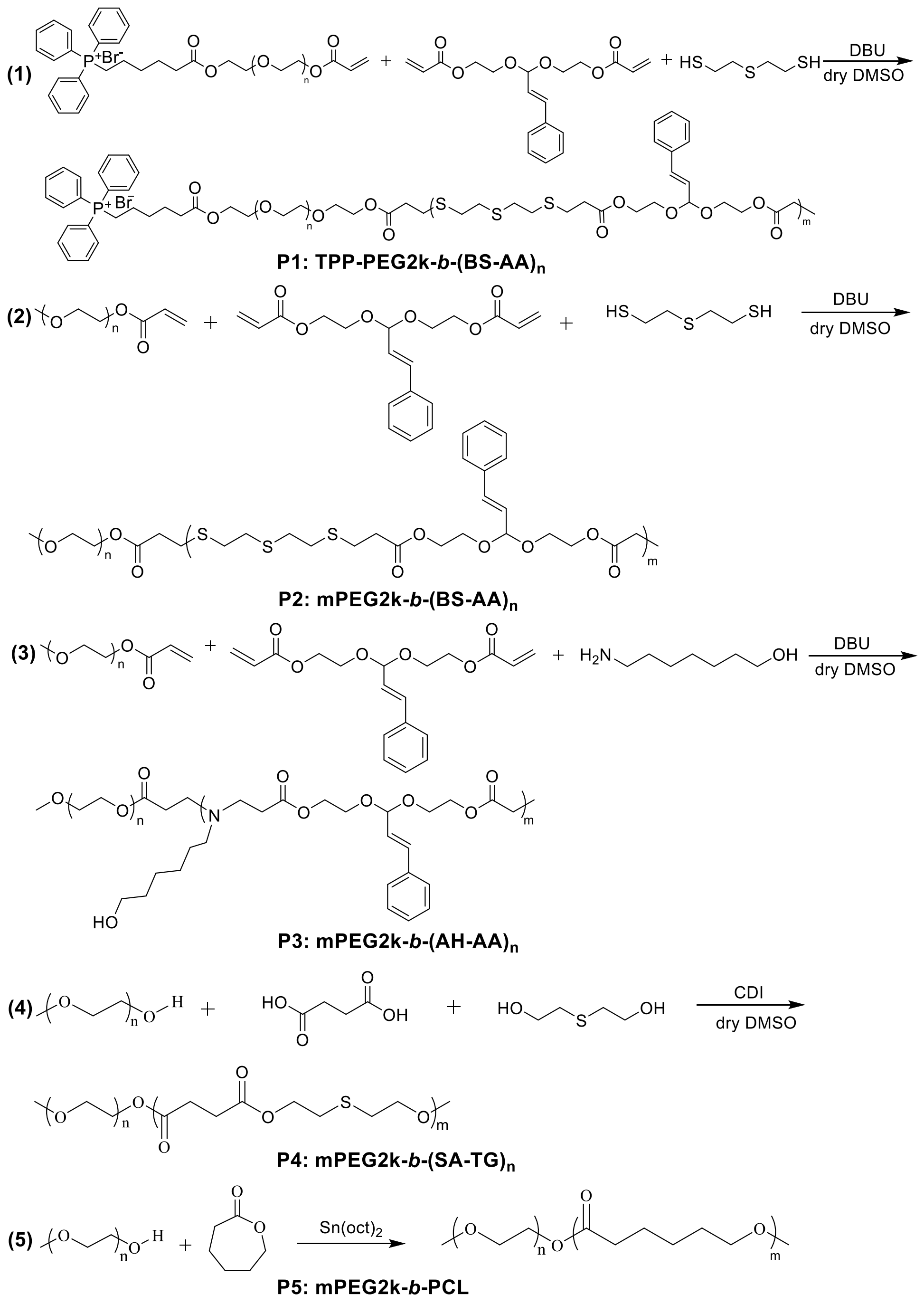

Scheme S2. Synthetic scheme of targeting mitochondria ROS/pH dual-responsive polymer P1 (A), ROS/pH dual-responsive polymer P2 (B), pH single-responsive polymer P3 (C)，ROS single-responsive polymer P4 (D), and ROS/pH inert control polymer P5 (E).

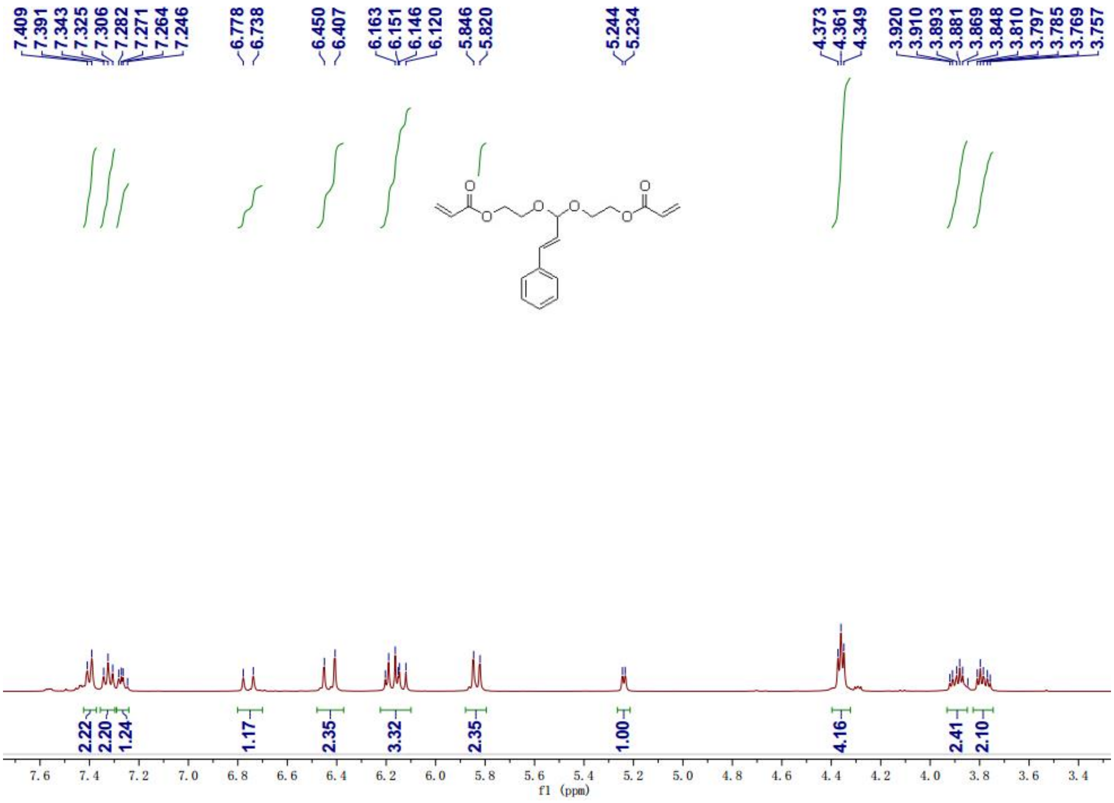

Figure S1. ^{1}H NMR of double-end alkenylated acetal AA.

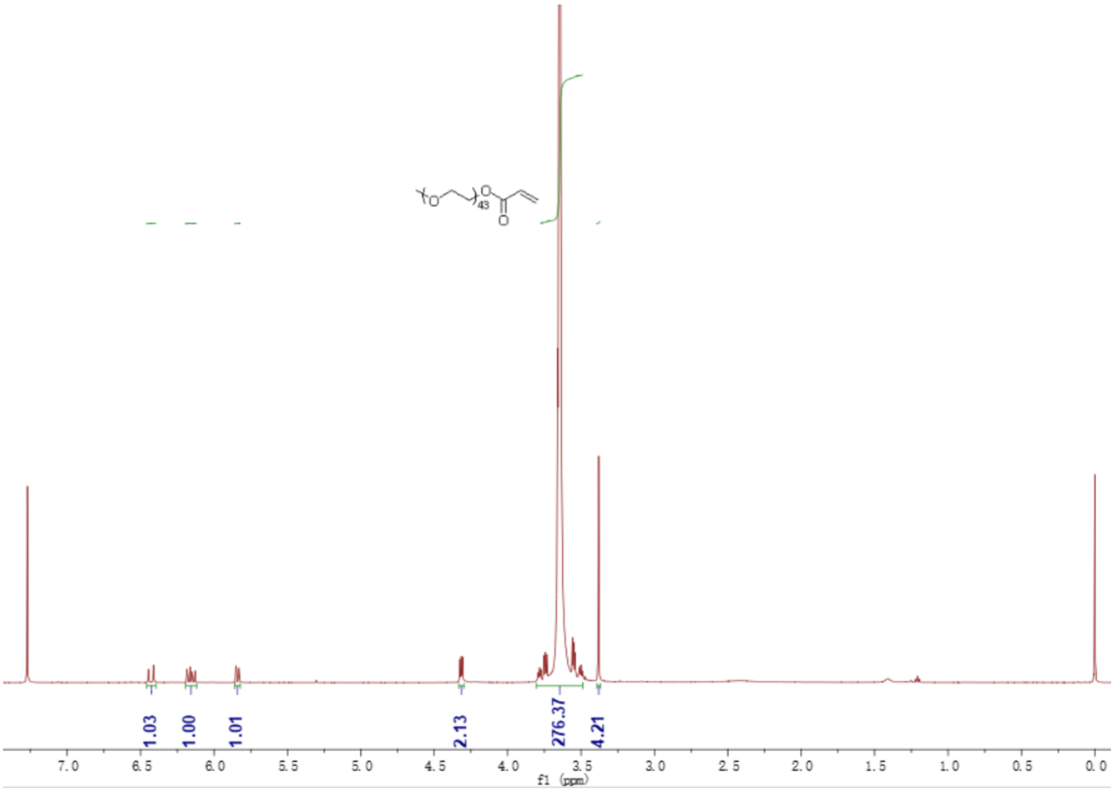

Figure S2. ^{1}H NMR of single-end alkenylated capping reagent mPEG2k-AC.

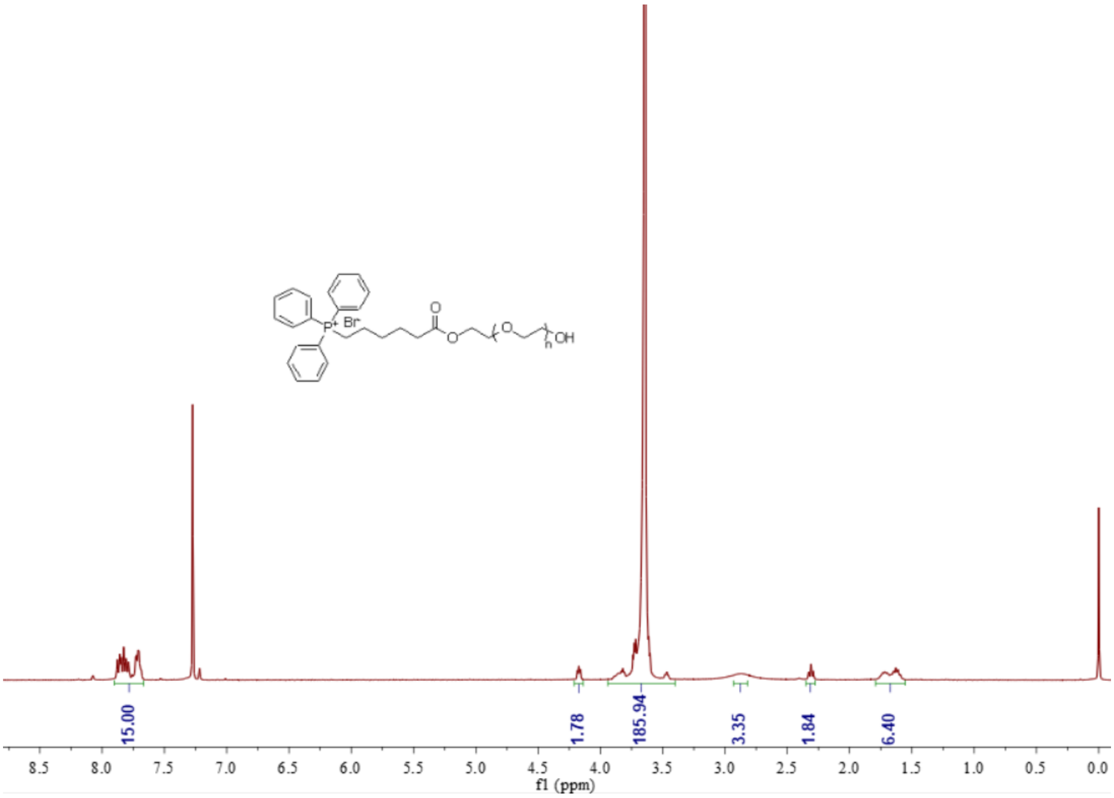

Figure S3. ^{1}H NMR of TPP-PEG2k.

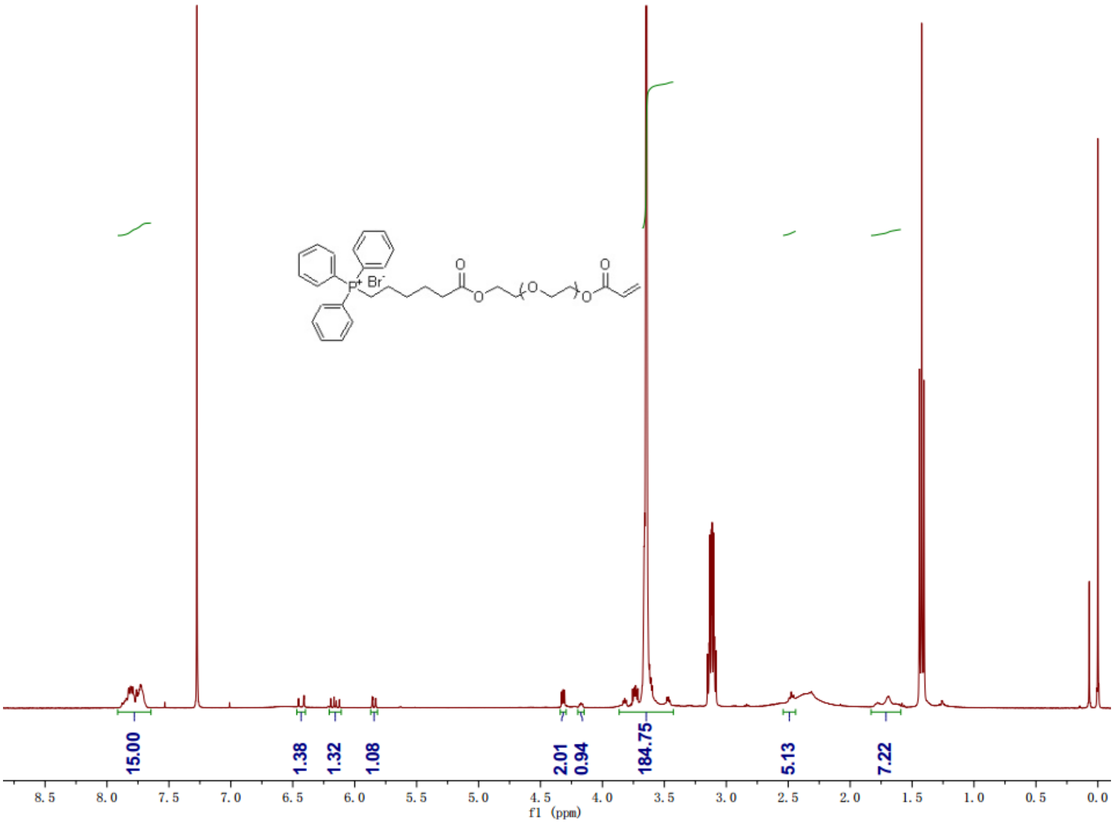

Figure S4. ^{1}H NMR of TPP-PEG2k-AC.

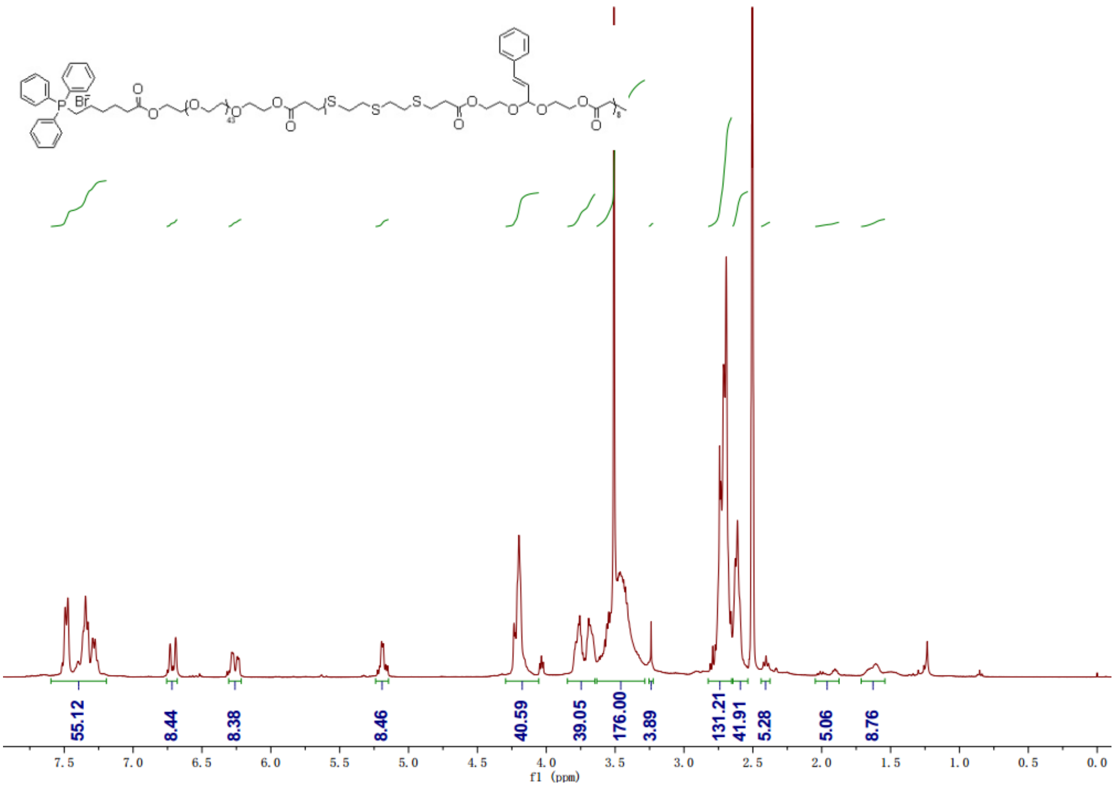

Figure S5. ^{1}H NMR of TPP-PEG-*b*-(BS-AT)$_{8}$.

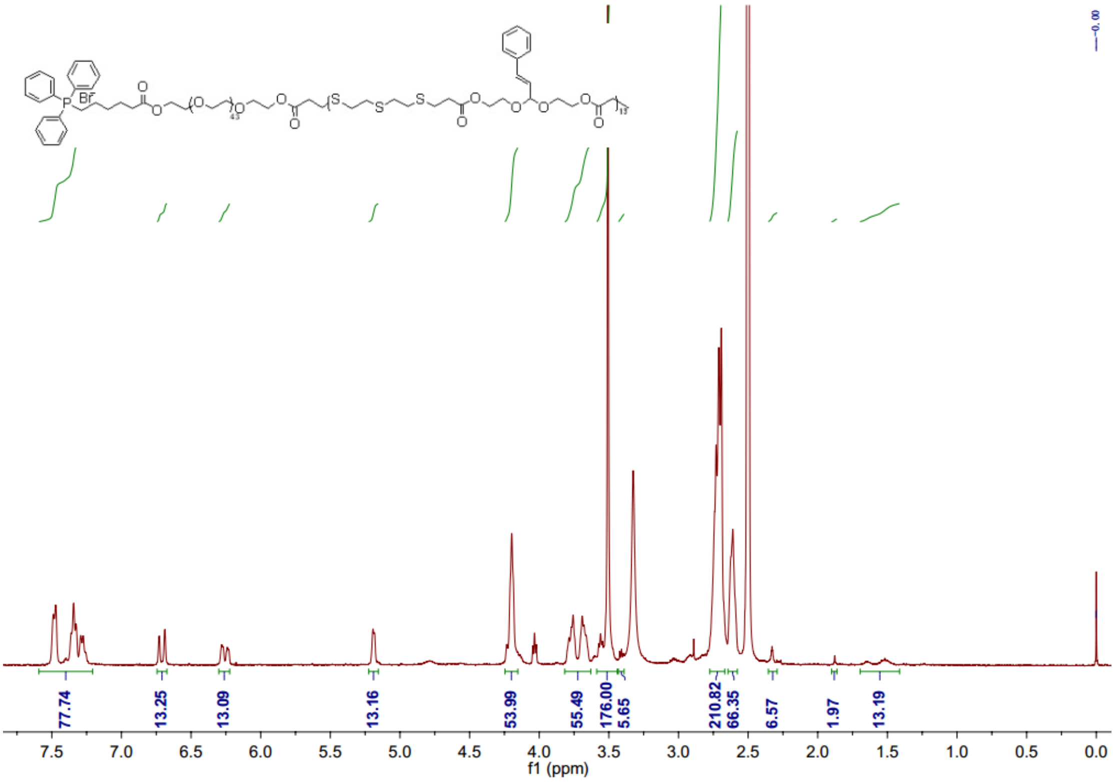

Figure S6. ^{1}H NMR of TPP-PEG-*b*-(BS-AT)$_{13}$.

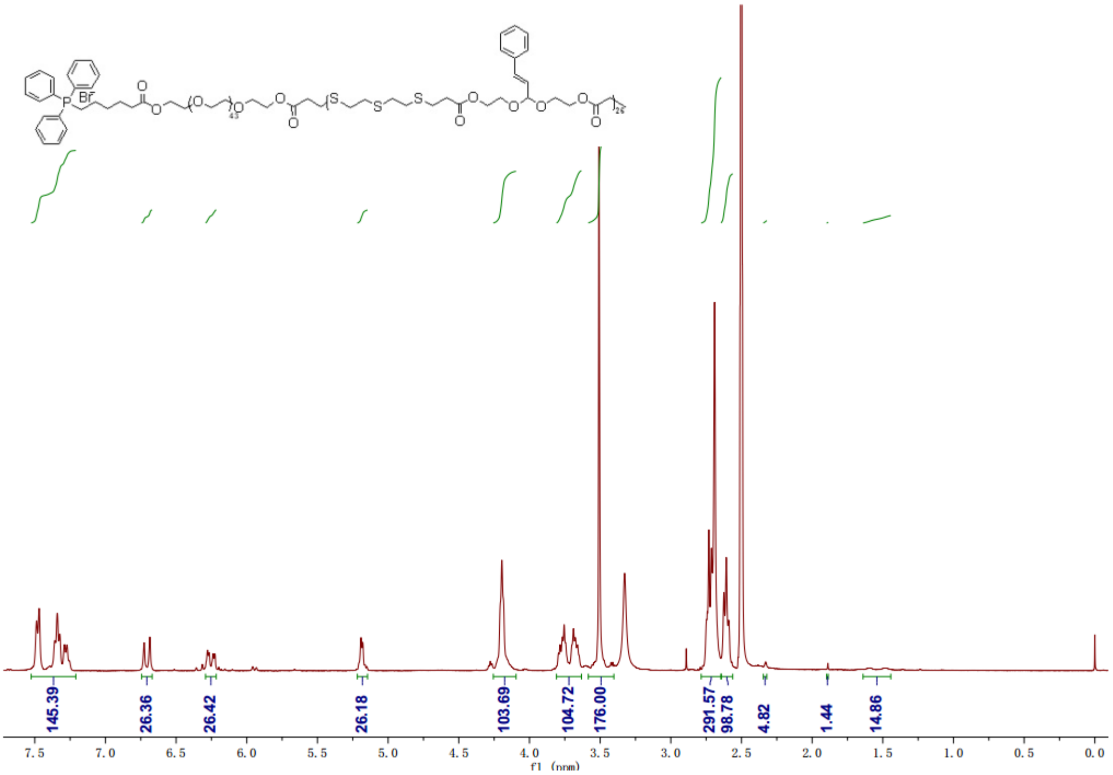

Figure S7. ^{1}H NMR of TPP-PEG-*b*-(BS-AT)$_{26}$.

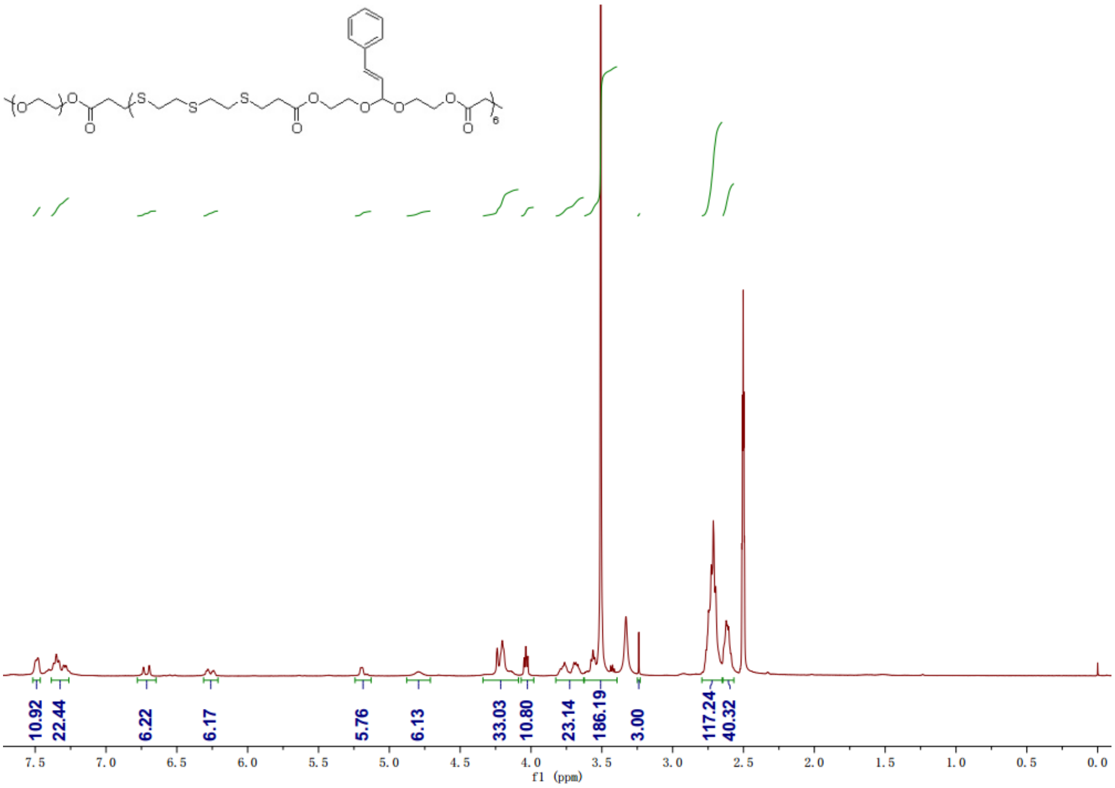

Figure S8. ^{1}H NMR of mPEG-*b*-(BS-AT)$_{6}$.

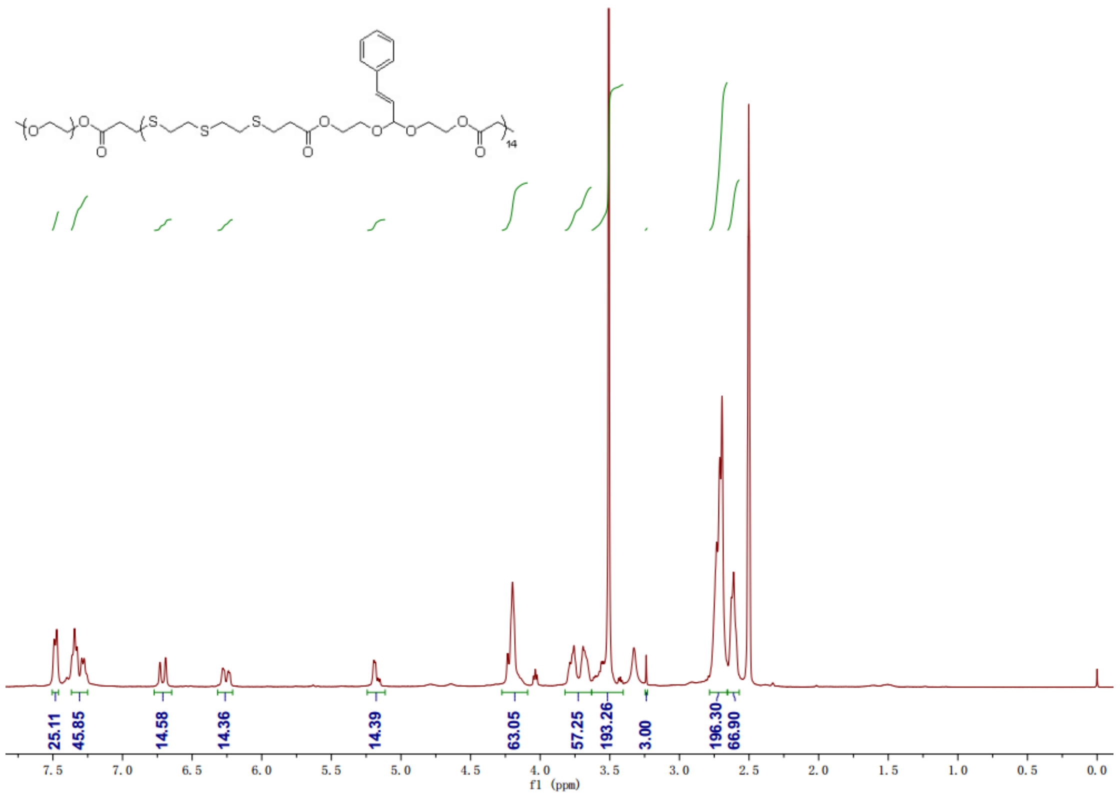

Figure S9. ^{1}H NMR of mPEG-*b*-(BS-AT)$_{14}$.

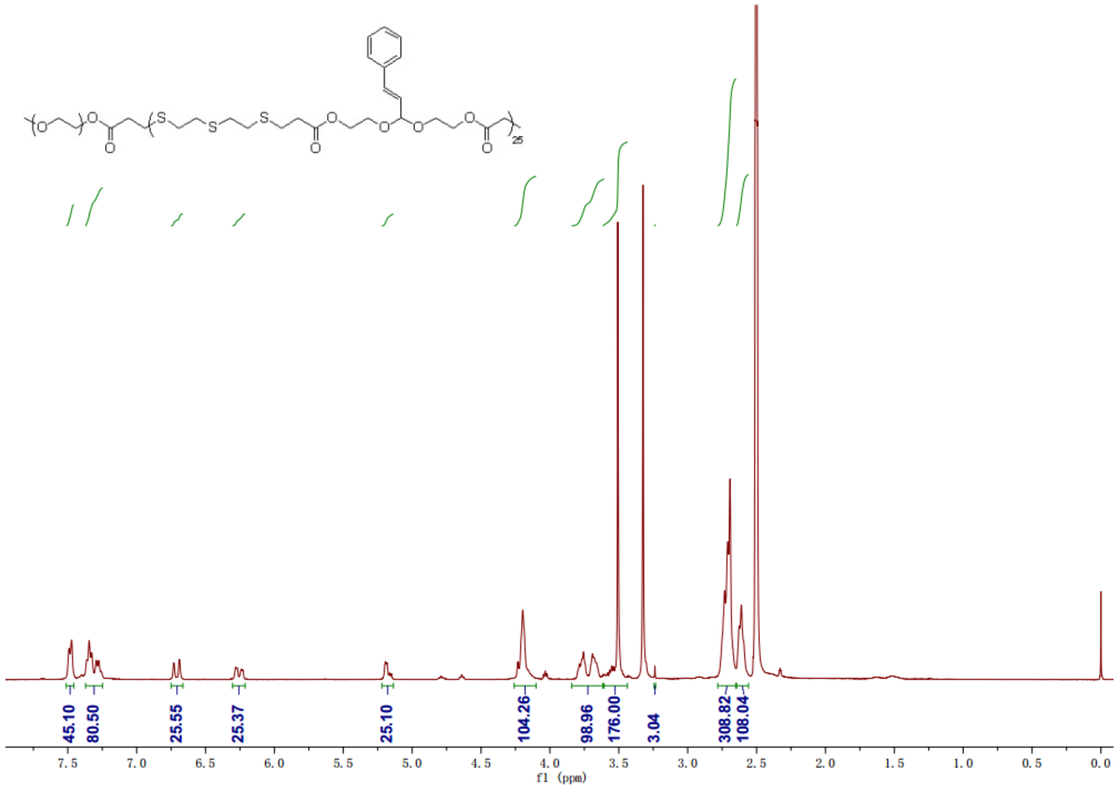

Figure S10. ^{1}H NMR of mPEG-*b*-(BS-AT)$_{25}$.

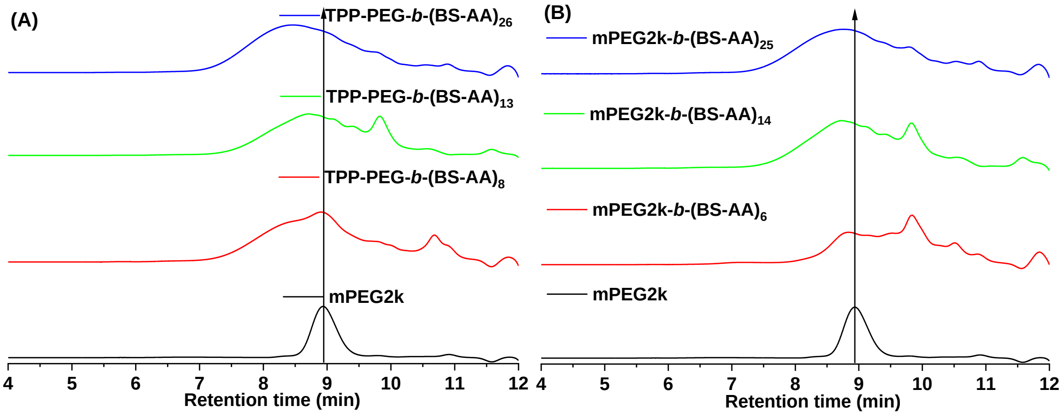

Figure S11. GPC traces of copolymer P1 and P2 with different number of repeating unit in DMF.

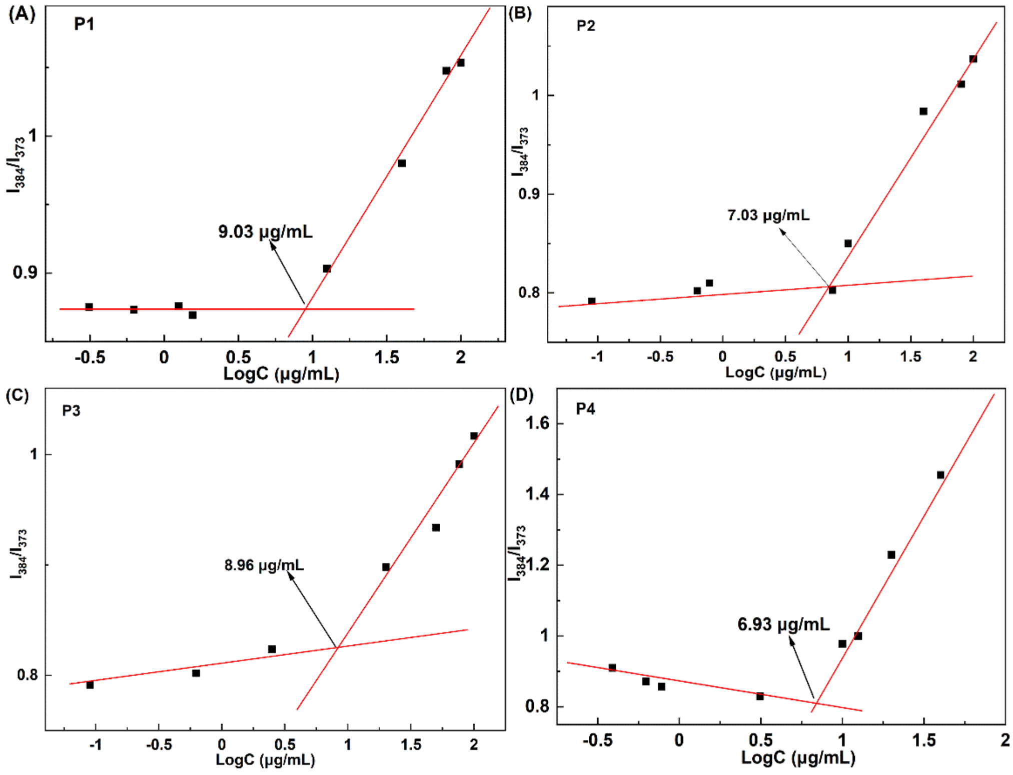

Figure S12. CMCs of TPP-PEG2k-*b*-(BS-AA)$_{13}$ P1 micelle (A), mPEG2k-*b*-(BS-AA)$_{14}$ P2 micelle (B), mPEG2k-*b*-(AH-AA)$_{10}$ P3 micelles (C), Rand mPEG2k-*b*-(SA-TG)$_{25}$ P4 micelles.

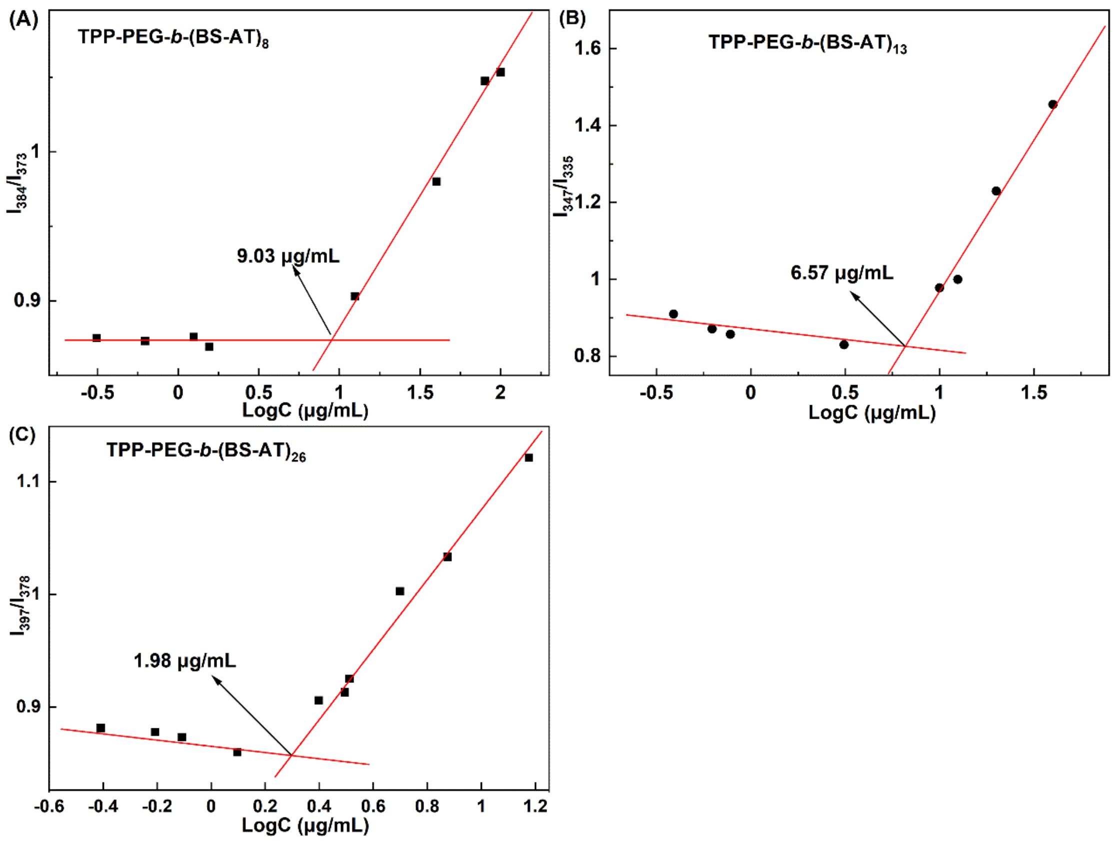

Figure S13. CMC of TPP-PEG2k-*b*-(BS-AA)$_8$ micelle (A), TPP-PEG2k-*b*-(BS-AA)$_{13}$ micelle (B), and TPP-PEG2k-*b*-(BS-AA)$_{26}$ micelle (C) .

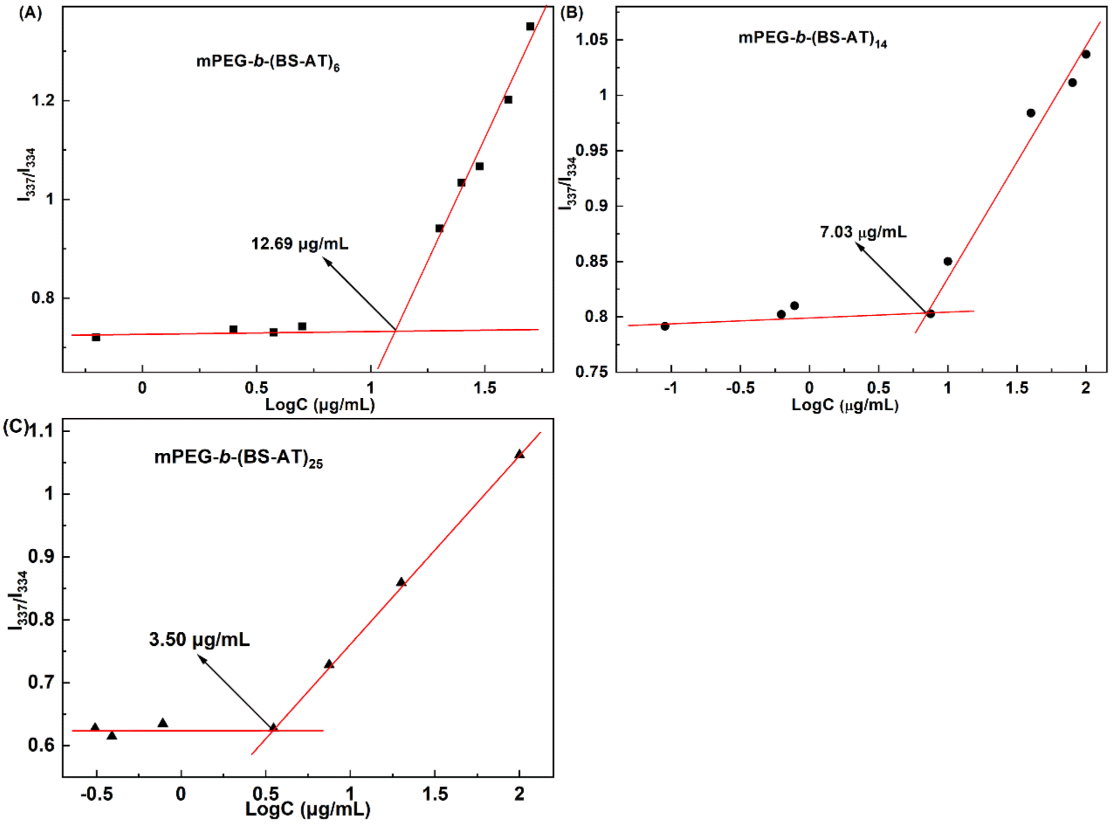

Figure S14. CMC of mPEG2k-*b*-(BS-AA)$_6$ micelle (A), mPEG2k-*b*-(BS-AA)$_{14}$ micelle (B), and mPEG2k-*b*-(BS-AA)$_{25}$ micelle (C) .

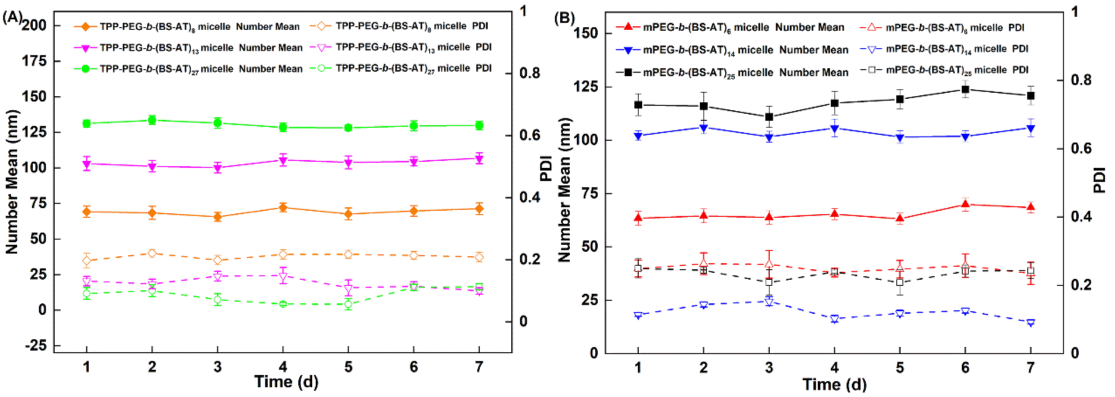

Figure S15. Stability of P1 and P2 micelle containing different number of repeating units measured by DLS at 25 °C in PBS (pH 7.4).

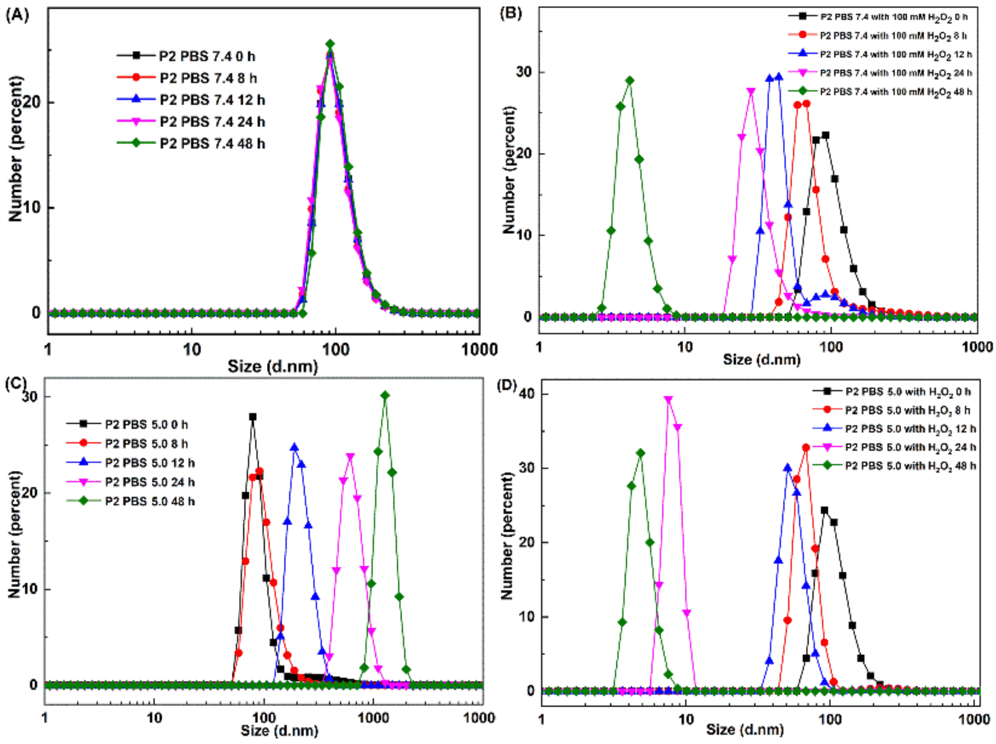

Figure S16 Size changes of mPEG2k-*b*-(BS-AA)$_{14}$ P2 micelle incubated with PBS 7.4 (A), PBS 7.4 with 100 mM H_2O_2 (B), PBS 5.0 (C), PBS 5.0 with 100 mM H_2O_2 (D) for different times.

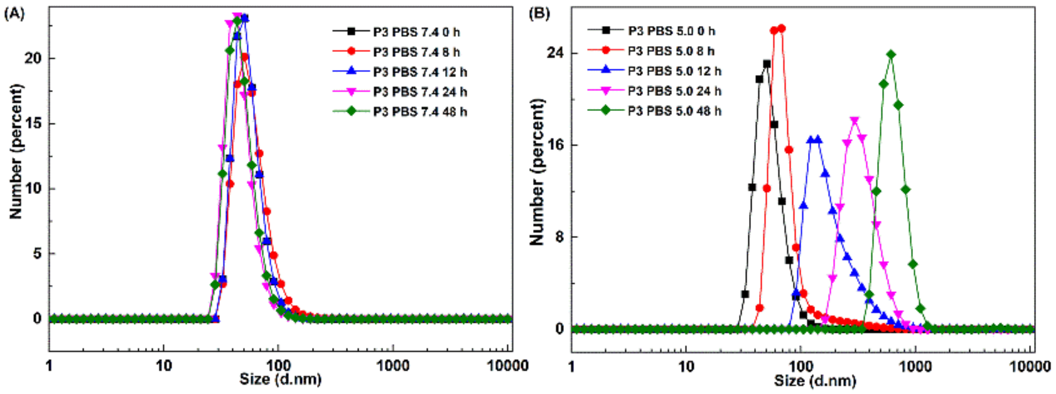

Figure S17 Size changes of mPEG2k-b-(AH-AA)10 P3 (C) micelle incubated with PBS 7.4 (A) and PBS 5.0 (B) for different times.

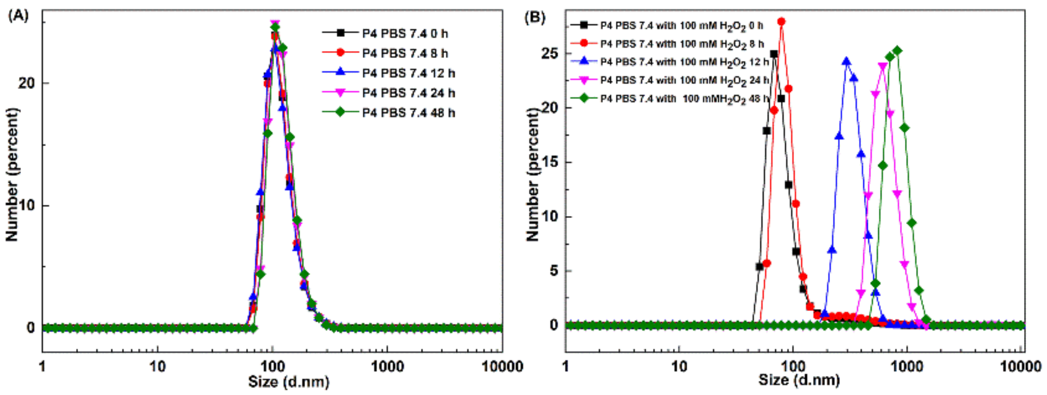

Figure S18 Size changes of mPEG2k-b-(SA-TG)$_{25}$ P4 micelle incubated with PBS 7.4 (A) and PBS 7.4 with 100 mM H_2O_2 (B) for different times.

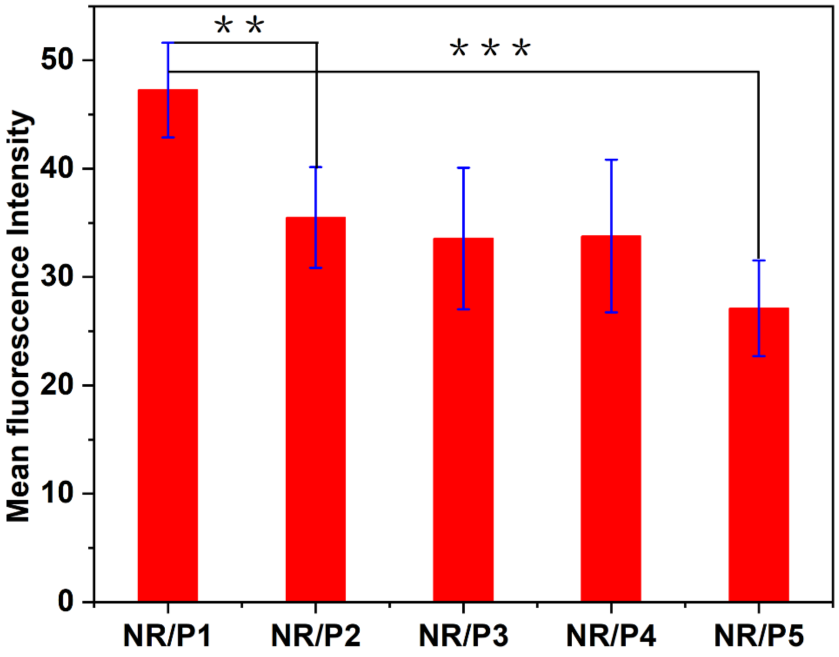

Figure S19. The semi-quantitative NR fluorescence intensity of sckov3 cells after treated with NR-loaded P1, P2, P3, P4, and P5 micelles for 6 h. Data are expressed as mean ± SD (n=7), statistical difference *P < 0.05, **P < 0.01, ***P < 0.001.
